# Postprandial metabolomics analysis reveals disordered serotonin metabolism in post-bariatric hypoglycemia

**DOI:** 10.1172/JCI180157

**Published:** 2024-09-12

**Authors:** Rafael Ferraz-Bannitz, Berkcan Ozturk, Cameron Cummings, Vissarion Efthymiou, Pilar Casanova Querol, Lindsay Poulos, Hanna Wang, Valerie Navarrete, Hamayle Saeed, Christopher M. Mulla, Hui Pan, Jonathan M. Dreyfuss, Donald C. Simonson, Darleen A. Sandoval, Mary-Elizabeth Patti

**Affiliations:** 1 Research Division, Joslin Diabetes Center, Boston, Massachusetts, USA.; 2 Harvard Medical School, Boston, Massachusetts, USA.; 3 Divsion of Endocrinology, Brigham and Women’s Hospital, Boston, Massachusetts, USA.; 4 Section of Nutrition, Department of Pediatrics, Division of Endocrinology, Diabetes, and Metabolism, University of Colorado Anschutz Medical Campus, Aurora, Colorado, USA.

**Keywords:** Endocrinology, Metabolism, Glucose metabolism

## Abstract

**BACKGROUND:**

Bariatric surgery is a potent therapeutic approach for obesity and type 2 diabetes but can be complicated by post-bariatric hypoglycemia (PBH). PBH typically occurs 1–3 hours after meals, in association with exaggerated postprandial levels of incretins and insulin.

**METHODS:**

To identify mediators of disordered metabolism in PBH, we analyzed the plasma metabolome in the fasting state and 30 and 120 minutes after mixed meal in 3 groups: PBH (*n* = 13), asymptomatic post–Roux-en-Y gastric bypass (post-RYGB) (*n* = 10), and nonsurgical controls (*n* = 8).

**RESULTS:**

In the fasting state, multiple tricarboxylic acid cycle intermediates and the ketone β-hydroxybutyrate were increased by 30%–80% in PBH versus asymptomatic. Conversely, multiple amino acids (branched-chain amino acids, tryptophan) and polyunsaturated lipids were reduced by 20%–50% in PBH versus asymptomatic. Tryptophan-related metabolites, including kynurenate, xanthurenate, and serotonin, were reduced 2- to 10-fold in PBH in the fasting state. Postprandially, plasma serotonin was uniquely increased 1.9-fold in PBH versus asymptomatic post-RYGB. In mice, serotonin administration lowered glucose and increased plasma insulin and GLP-1. Moreover, serotonin-induced hypoglycemia in mice was blocked by the nonspecific serotonin receptor antagonist cyproheptadine and the specific serotonin receptor 2 antagonist ketanserin.

**CONCLUSION:**

Together these data suggest that increased postprandial serotonin may contribute to the pathophysiology of PBH and provide a potential therapeutic target.

**FUNDING:**

National Institutes of Health (NIH) grant R01-DK121995, NIH grant P30-DK036836 (Diabetes Research Center grant, Joslin Diabetes Center), and Fundação de Amparo à Pesquisa do Estado de São Paulo grant 2018/22111-2.

## Abstract 2

Metabolomics analysis reveal reduced fasting and elevated postprandial serotonin levels in individuals with hypoglycemia after bariatric surgery.

## Graphical abstract

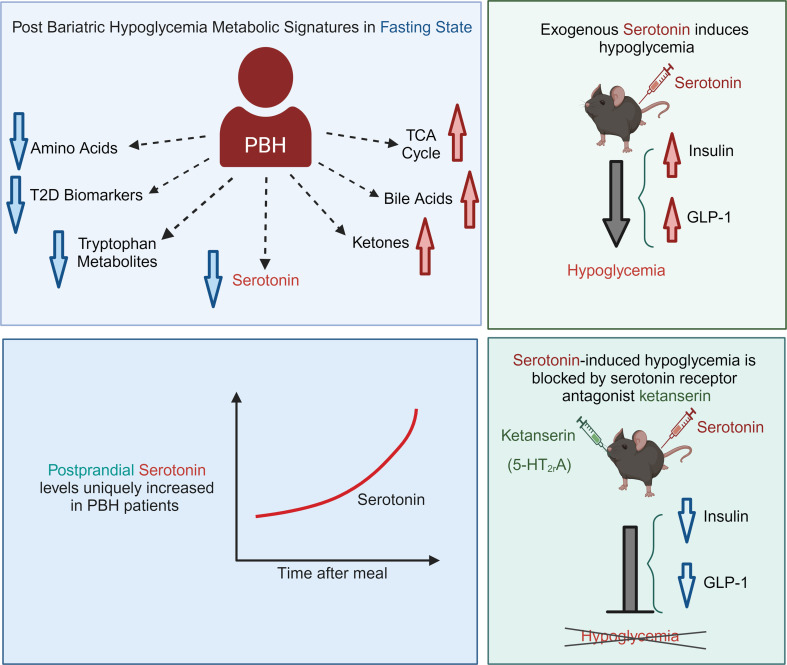

## Introduction

Bariatric and upper gastrointestinal surgery exerts profound improvements in glucose metabolism and can even induce remission of type 2 diabetes (T2D) (1, 2), thus demonstrating the importance of the gastrointestinal tract as a key regulator of systemic glucose metabolism. While the mechanisms responsible for these potent effects remain incompletely understood, contributors include altered postprandial nutrient delivery to the intestine, increased secretion of glucagon-like peptide 1 (GLP-1) by intestinal L cells, increased insulin secretion, intestinal mucosal adaptations, alterations in the gut-brain axis, and changes in bile acid content and composition (3, 4).

Despite the metabolic benefits of bariatric surgery, one increasingly recognized and often severe complication of bariatric surgery is postprandial hypoglycemia, termed post-bariatric hypoglycemia (PBH). PBH is characterized by hypoglycemia with neuroglycopenia, typically developing 1–3 years or more postoperatively. Hypoglycemia is most commonly recognized 1–3 hours after meals (5), potentially related to the even greater increases in meal-stimulated GLP-1 and insulin secretion in patients with PBH as compared with unaffected post-surgical patients (5). Indeed, incretin hormone action can reduce postprandial insulin secretion and improve hypoglycemia (6). However, hypoglycemia may also occur with activity and during mid-nocturnal hours (7), suggesting additional mechanisms unique to those affected by PBH. These include increased insulin-independent glucose uptake (8) and increased levels of the intestinally derived hormone FGF-19 (9). Given that this disorder represents a model of “extreme” modification of glucose metabolism in response to bariatric/metabolic surgery, understanding the metabolic pathways perturbed in PBH may inform our understanding of intestinal regulation of glucose homeostasis and uncover new therapeutic targets for PBH and, more broadly, for nonsurgical approaches to improve systemic glucose metabolism.

We hypothesized that additional perturbations in the metabolome unique to PBH could modulate key substrates for gluconeogenesis and/or other pathways contributing to hypoglycemia. We thus performed untargeted semiquantitative metabolomic analysis of plasma samples collected in the fasting state and at 30 and 120 minutes after administration of a liquid mixed meal in 3 groups of human volunteers: individuals with PBH, those without hypoglycemia after Roux-en-Y gastric bypass (RYGB) (asymptomatic post-RYGB), and overweight/obese individuals without history of gastrointestinal surgery. We report that metabolic signatures in PBH suggest an accelerated metabolic return to the fasting state, as evidenced by increases of multiple tricarboxylic acid (TCA) cycle intermediates and the ketone β-hydroxybutyrate. In addition, PBH is characterized by reduction in fasting plasma levels of amino acids (AAs), multiple lipid species, and tryptophan intermediates including kynurenate, xanthurenate, and serotonin. Moreover, plasma serotonin levels are uniquely increased in the postprandial state in patients with PBH. We demonstrate that serotonin administration in mice increases insulin and GLP-1 levels and reduces plasma glucose; these effects are blocked by the nonspecific serotonin antagonist cyproheptadine. Moreover, the serotonin receptor 2 antagonist ketanserin abolished the impact of serotonin to induce hypoglycemia. Together these data suggest that increased postprandial serotonin may contribute to the pathophysiology and provide a new therapeutic target for PBH.

## Results

### Clinical parameters.

Demographic information was self-reported by participants and is provided in Supplemental Table 1 (supplemental material available online with this article; https://doi.org/10.1172/JCI180157DS1). Participants were a subset from a prior study (5) for whom pristine residual samples were available for analysis. These included 13 individuals with PBH (11 female, 2 male; mean age 48 ± 11, BMI 30.6 ± 4.9, 3.1 ± 1.6 years post-RYGB), 10 individuals post-RYGB without symptomatic hypoglycemia (Asx) (8 female, 2 male; age 48 ± 8, BMI 29.9 ± 4.4, 2.6 ± 1.0 years post-RYGB), and 8 overweight/obese individuals without T2D and without history of gastrointestinal surgery (Ow/Ob) (5 female, 2 male; age 48 ± 9, BMI 41.8 ± 10.8). Hemoglobin A_1c_ did not differ between groups. Fasting glucose, insulin, triglycerides, homeostatic model assessment for insulin resistance (HOMA-IR), and Matsuda index were significantly lower, and HDL significantly higher, in both surgical groups as compared with nonsurgical Ow/Ob individuals, consistent with improved glucose metabolism and insulin sensitivity postoperatively, but did not differ between PBH and Asx.

During mixed meal testing, nonsurgical participants displayed the expected meal-related rise in glucose with return to baseline by 120 minutes. However, patterns were distinct in the asymptomatic post-surgical patients, with greater glycemic excursions versus controls; participants with PBH had lower glucose levels at 30 minutes versus asymptomatic (128 ± 32.3 mg/dL vs. 162 ± 38.3 mg/dL at 30 minutes, *P* = 0.019; Supplemental Figure 1A). At 2 hours, PBH and Asx glucose levels were similar, but significantly lower than in nonsurgical individuals (PBH 68.7 ± 11.3 mg/dL; Asx 70.3 ± 8.6 mg/dL; Ow/Ob 98.3 ± 11.4 mg/dL; *P* < 0.001 for both PBH vs. Ow/Ob and Asx vs. Ow/Ob).

Fasting insulin and C-peptide were reduced in PBH and Asx groups compared with Ow/Ob, but did not differ between PBH and Asx. After meal ingestion, insulin and C-peptide levels increased markedly in PBH, returning to baseline by 2 hours (Supplemental Figure 1, B and C). Plasma GLP-1 was significantly increased in both surgical groups, with even higher levels in PBH at 30 minutes (209.3 vs. 125.8 pmol/L for Asx, *P* < 0.001; Supplemental Figure 1D). Gastric inhibitory polypeptide (GIP) levels did not differ between PBH and Asx, but GIP levels in Asx were higher than those in Ow/Ob at 30 minutes (*P* < 0.01) (Supplemental Figure 1E). Glucagon levels did not differ in the fasting state but were higher after meals in both surgical groups (Supplemental Figure 1F). Plasma cortisol did not differ between groups (Supplemental Figure 1G).

### Metabolite patterns differ in PBH.

**Figure 1:**
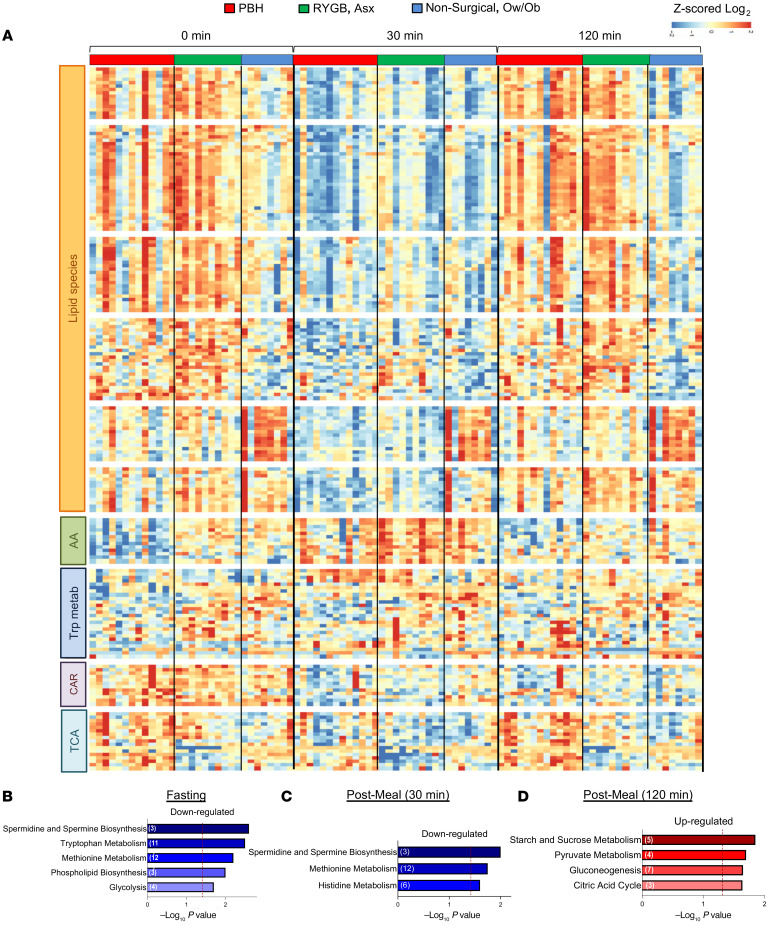
Metabolite abundance in individuals with PBH, Asx, and Ow/Ob in the fasting state and at 30 and 120 minutes after mixed meal ingestion. (**A**) Heatmap of *z*-scored log_2_-transformed metabolite abundance for the top 190 most altered metabolites between groups, with unsupervised hierarchical clustering of rows. Cluster annotations include lipid species (clusters 1–6), amino acids (AA; cluster 7), tryptophan metabolites (cluster 8), carnitines (CAR; cluster 9), and tricarboxylic acid (TCA) cycle metabolites (cluster 10). (**B**–**D**) Pathway enrichment analysis for PBH versus Asx individuals in the fasting state and 30 and 120 minutes after meal ingestion, respectively. Downregulated pathways are colored blue, while upregulated pathways are colored red. The –log_10_ *P* value for enrichment is represented on the *x* axis; the vertical line indicates nominal *P* less than 0.05.

Unsupervised hierarchical clustering of the 189 metabolites detected at all 3 time points and with significant FDR less than 0.05 (*F* test) is presented in the heatmap in Figure 1A, with a full list of cluster components provided in Supplemental Table 2 and full statistical analysis in Supplemental Table 3. As expected, ingestion of the mixed meal in nonsurgical controls resulted in decreased abundance of multiple lipid species and increased abundance of AAs at 30 minutes, with return to baseline at 120 minutes.

Exploratory analysis of these 189 metabolites revealed that 158 metabolites were altered in Asx individuals versus nonsurgical controls, reflecting the impact of surgery (FDR < 0.25). Sixty-two metabolites were altered in PBH versus Asx post-surgical individuals, and 178 metabolites were differentially abundant in both comparisons. Interestingly, 19 of these 178 had opposite directionality between PBH and Asx, including adenine, oxalate, and hydroxyphenylpyruvate (Supplemental Figure 2, A–C). These findings support the concept that PBH is not simply an “extreme” example of post-surgical metabolic perturbations.

Pathway analysis was performed using the Fry function of the rotation gene set test (ROAST) in the limma R package. Multiple pathways were enriched (FDR < 0.25) in the comparison of PBH and Asx post-surgical groups (Figure 1, B–D). In the fasting state, pathways downregulated in PBH included spermidine/spermine biosynthesis, tryptophan and methionine metabolism, phospholipid synthesis, and glycolysis (Figure 1B). After meals, both spermidine/spermine and methionine metabolism pathways remained downregulated at 30 minutes (Figure 1C). By 2 hours, enriched pathways were upregulated in PBH, including starch/sucrose and pyruvate metabolism, gluconeogenesis, and citric acid cycle pathways (Figure 1D). Additional comparisons are presented in Supplemental Figure 3 (PBH vs. Ow/Ob) and Supplemental Figure 4 (Asx vs. Ow/Ob).

### Correlation of metabolites with glucose.

As expected, both AAs and lipids were highly correlated with glucose levels. The top metabolites that correlated with glucose in the fasting state (FDR < 0.05) (Supplemental Table 4) were AAs (Phe, Tyr, Met, Leu, Trp, His, Ala, and Asn), with similar patterns at 30 minutes. Additional metabolites correlating with glucose in the fasting state included NMMA (*N*-methylmalonamic acid), carnitine, UDP (uridine-5′-diphosphate), and oxalate. At 30 minutes, metabolites correlating with glucose included AAs (Leu, Met, Ile, Phe, Tyr, Gln, Pro, Arg, Trp, Gly, Ser, His, Val, Lys, Thr), citrulline, choline, NMMA, and ornithine, and the triglycerides C56:1, C48:0, and C50:0 (FDR < 0.05). There were no metabolites significantly correlating (FDR < 0.05) with glucose at 120 minutes (Supplemental Table 4).

Given that hypoglycemia typically develops 2–3 hours after meals, we asked whether metabolites present in the fasting state correlated with post-meal glucose. While relationships were not as robust as for the matched-time analyses, fasting levels of 17 metabolites were positively correlated with glucose at 120 minutes, including lactate, AAs (Gln, Pro, Trp, Tyr, betaine), carnitine, and multiple lipids (lysophosphatidylcholine [LPC] 18:0, 18:2, 20:3, and 22:6; lysophosphatidylethanolamine [LPE] 18:1 and 22:6) (FDR < 0.25) (Supplemental Figure 5).

### Plasma levels of TCA cycle intermediates are increased in PBH.

**Figure 2:**
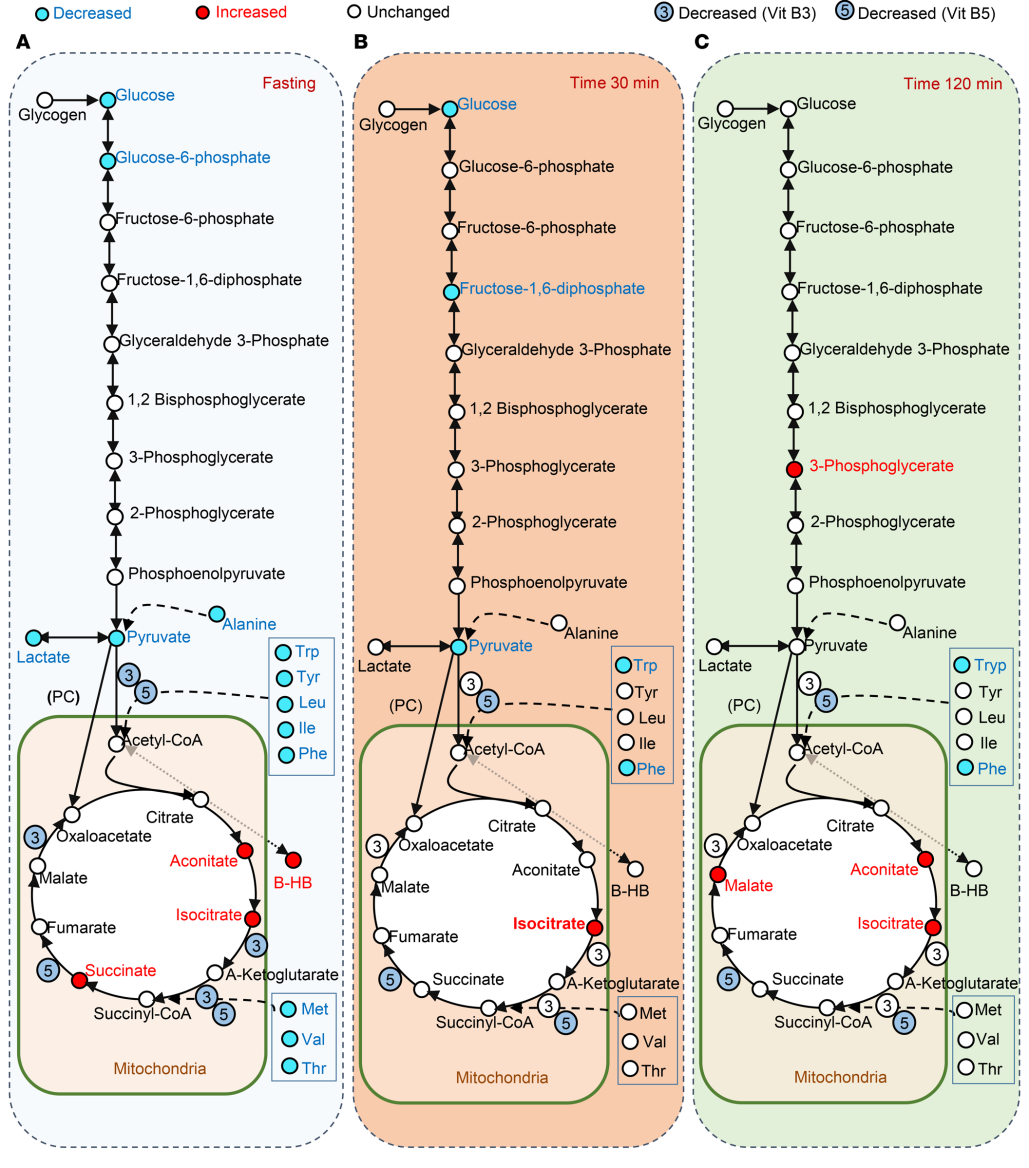
Model demonstrating the metabolites of the glycolysis, gluconeogenesis, and TCA cycle pathways during mixed meal tolerance test. Individuals with PBH and Asx are compared in the fasting state (**A**) and at 30 minutes (**B**) and 120 minutes (**C**) after mixed meal ingestion. Metabolites are colored blue if decreased (nominal *P* < 0.05), red if increased (nominal *P* < 0.05), and white if unchanged in PBH versus Asx.

Hypoglycemia ultimately may reflect interaction between excessive insulin secretion, inadequate counterregulatory hormone levels and/or action, reduced glycogen stores, and inadequate substrate or activity within gluconeogenic pathways. As noted above, multiple AAs were reduced in PBH, potentially contributing to reduced formation of intermediates such as pyruvate (alanine), acetyl-CoA (tryptophan, tyrosine, leucine, isoleucine, and phenylalanine), and succinyl-CoA (methionine, valine, and threonine). We interrogated metabolite patterns in central glucose metabolic pathways, projecting differences in PBH onto a pathway map at each time point (Figure 2).

In the fasting state (Figure 2A), PBH was characterized not only by decreased levels of glucose (20% lower, *P* = 0.001, FDR = 0.04) but also by decreases in gluconeogenesis intermediates such as glucose-6-phosphate (40% reduction, *P* = 0.02, FDR = 0.18) and pyruvate (80% lower, *P* < 0.0001, FDR = 0.003). Conversely, TCA cycle intermediates such as aconitate, isocitrate, and succinate were increased by 30% in PBH versus Asx in the fasting state (*P* < 0.05 for all, FDR < 0.25). Moreover, the ketone β-hydroxybutyrate was increased by 80%, as noted above (fold change = 1.8, *P* = 0.04, FDR < 0.25).

At 30 minutes after meals, only glucose and pyruvate remained lower (20% and 50% lower, *P* < 0.01, FDR < 0.1), and isocitrate remained higher (30% higher, *P* = 0.009, FDR = 0.14), in PBH versus Asx (Figure 2B). At 2 hours, 3-phosphoglycerate was 2-fold higher (*P* = 0.002, FDR = 0.002), while several TCA intermediates were again higher in PBH, including 30%–40% increases in aconitate, isocitrate, and malate (all *P* < 0.01, FDR < 0.01) (Figure 2C).

### Lipid, ketone, and bile acid metabolism is perturbed in PBH.

Multiple lipid species were decreased in both surgical groups at 30 minutes, consistent with postprandial rises in insulin secretion. Additional lipid species differentially abundant in PBH versus Asx included LPE 20:4 and C22:6, LPC C22:6, and triacylglycerol (TAG) C50:5, C54:5, and C56:7, all of which were decreased by 30%–50% in PBH versus Asx (*P* < 0.05, FDR < 0.25) (Supplemental Figure 6, A–F). By contrast, C56:1 TAG was markedly increased in PBH versus Asx at 30 and 120 minutes (29- and 59-fold higher, *P* = 0.01, FDR < 0.25 for both; Supplemental Figure 6G). In agreement, abundance of C56:1 TAG was inversely correlated with glucose levels at both 30 (*r* = –0.67, *P* = 0.01) and 120 minutes (*r* = –0.65, *P* = 0.02) in PBH, but not in the fasting state.

Ketones, produced in the liver from 2-carbon products of β-oxidation, can be utilized by some tissues as an alternative fuel when glucose availability is limited, as with prolonged fasting. Interestingly, the ketone β-hydroxybutyrate was increased in the fasting state by 80% in PBH versus Asx (*P* = 0.04, FDR < 0.25) and more than 3-fold in comparison with Ow/Ob (*P* < 0.001, FDR = 0.01). These differences remained at 120 minutes (Supplemental Figure 6H).

Bile acids have been proposed as mediators of glycemic improvement after RYGB (4), potentially via effects to increase both incretin hormones and FGF-19 (4, 10). Fasting levels of the conjugated bile acid taurocholate were 2.5-fold higher in PBH versus Asx (*P* = 0.03, FDR = 0.22) (Supplemental Figure 6I). However, there were no significant differences in fasting or postprandial levels of other measured bile acids in PBH versus asymptomatic post-surgical participants (4, 9, 11).

### Biomarkers of insulin resistance and T2D are decreased in PBH.

Previous studies have identified metabolite biomarkers of insulin resistance and risk for T2D (12, 13). These include AAs (branched-chain AAs, Phe, and Tyr), and triglyceride species characterized by lower carbon number and double bond content (14). Indeed, post-surgical individuals had reductions in these biomarker AAs and lipids, consistent with improved metabolism after RYGB, with further reductions in PBH. Moreover, α-hydroxybutyrate, an organic acid derived from α-ketobutyrate (produced by AA catabolism) and associated with insulin resistance, was 30% lower in PBH at both fasting (*P* = 0.03, FDR = 0.21) and 30 minutes after meals (*P* = 0.03, FDR = 0.22), as compared with Asx (Supplemental Figure 7A). Likewise, 2-aminoadipate, a product of lysine degradation associated with insulin resistance (13, 15), was also 6-fold lower in PBH versus Asx (*P* = 0.001, FDR = 0.04) in the fasting state (Supplemental Figure 7B).

### Levels of B-complex vitamins are reduced in PBH.

B-complex vitamins play important roles as cofactors for key reactions in cellular metabolism. Several water-soluble B vitamins, including niacinamide (B_3_) and pantothenate (B_5_), were decreased 2-fold in the fasting state in PBH versus Asx (*P* < 0.05 for all, FDR < 0.25). Pantothenate was also 2-fold lower at 30 and 120 minutes (*P* < 0.01 for both, FDR < 0.05) (Supplemental Figure 7, C and D). Moreover, fasting glucose was positively correlated with levels of both vitamins B_3_ (fasting: *r* = 0.48, *P* = 0.02, FDR 0.11) and B_5_ (fasting: *r* = 0.52, *P* = 0.009, FDR = 0.07; 30 minutes: *r* = 0.49, *P* = 0.017, FDR = 0.14).

### AA levels are reduced in PBH in the fasting state.

**Figure 3:**
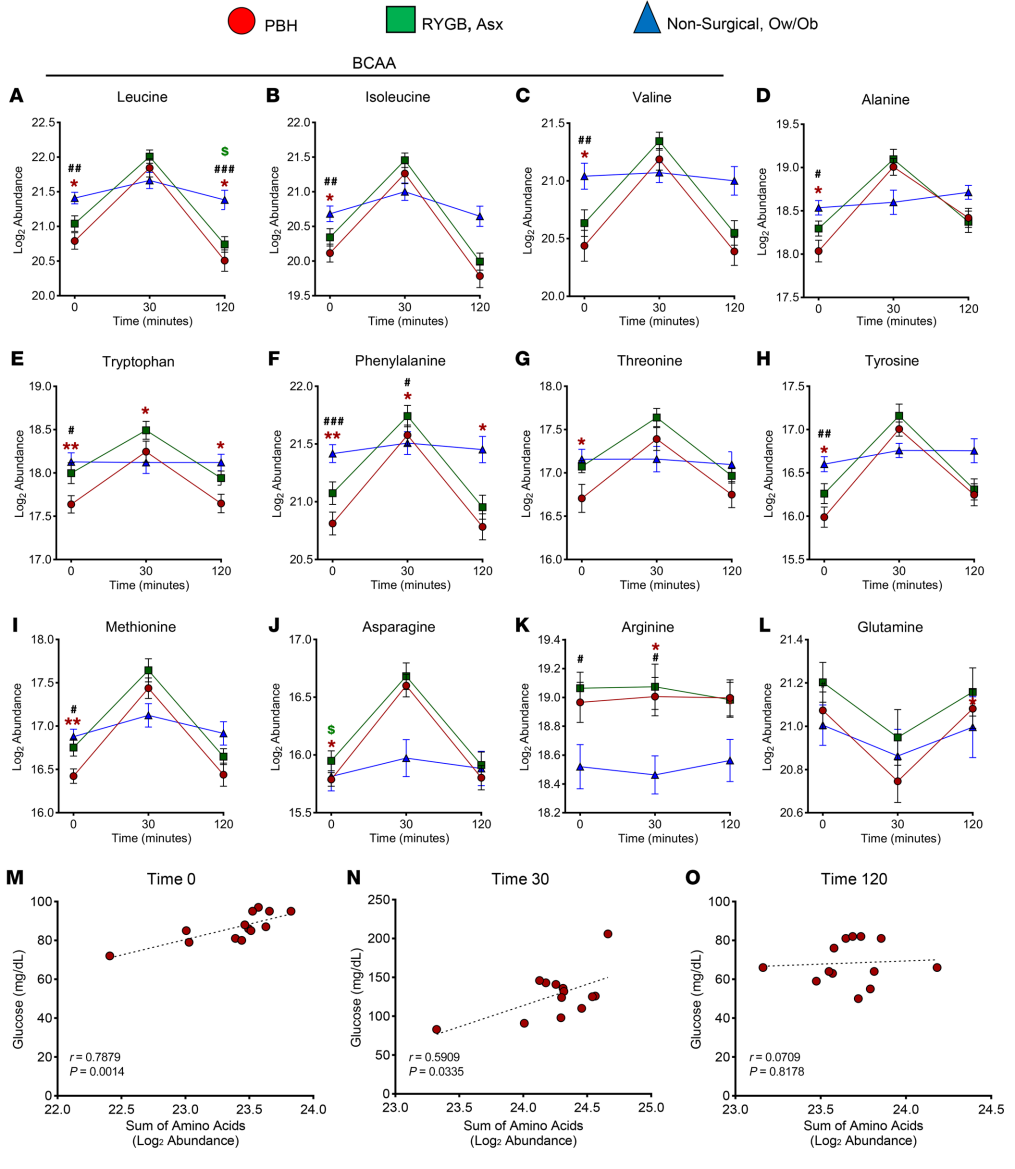
Abundance of 12 plasma AAs in PBH, Asx, and Ow/Ob individuals in the fasting state and at 30 and 120 minutes after mixed meal ingestion. (**A**–**L**) Abundance of leucine (**A**), isoleucine (**B**), valine (**C**), alanine (**D**), tryptophan (**E**), phenylalanine (**F**), threonine (**G**), tyrosine (**H**), methionine (**I**), asparagine (**J**), arginine (**K**), and glutamine (**L**). (**M**–**O**) Correlation between glucose levels and sum of AA abundance in the fasting state (red, **M**) and at 30 minutes (red, **N**) and 120 minutes (red, **O**) after meal. Data are mean ± SD. *^,#,$^*P* < 0.05; **^,##^*P* < 0.01; ^###^*P* < 0.001. Two-way ANOVA with Tukey’s multiple-comparison test was performed.

In the fasted state, 10 AAs were significantly reduced in PBH as compared with both asymptomatic and nonsurgical groups. Leucine, isoleucine, valine, alanine, tryptophan, phenylalanine, threonine, tyrosine, methionine, and asparagine were reduced by 17%–29% (*P* < 0.05, FDR < 0.25) in PBH versus Asx and Ow/Ob groups (Figure 3, A–J). After meal ingestion, AA levels increased markedly in both surgical groups, but only leucine, tryptophan, and phenylalanine remained lower in PBH as compared with Asx. AA levels for each participant are presented in Supplemental Figure 8. Notably, the reductions in AAs in PBH included both essential and non-essential AAs, and both gluconeogenic (e.g., Ala) and ketogenic (e.g., Leu) AAs. Arginine abundance was increased in both PBH and Asx compared with Ow/Ob (Figure 3K), while glutamine did not differ between groups (Figure 3L). The sum of all AAs was strongly positively correlated with glucose in the fasting state (*r* = 0.78, *P* = 0.001) and at 30 minutes (*r* = 0.59, *P* = 0.033); these correlations did not remain at 120 minutes (*r* = 0.07, *P* = 0.82) (Figure 3, M–O). The top-ranking specific AAs correlated with glucose levels both in the fasting state and at 30 minutes are provided in Supplemental Figure 9.

### Serotonin levels are uniquely increased in the postprandial state in individuals with PBH.

**Figure 4:**
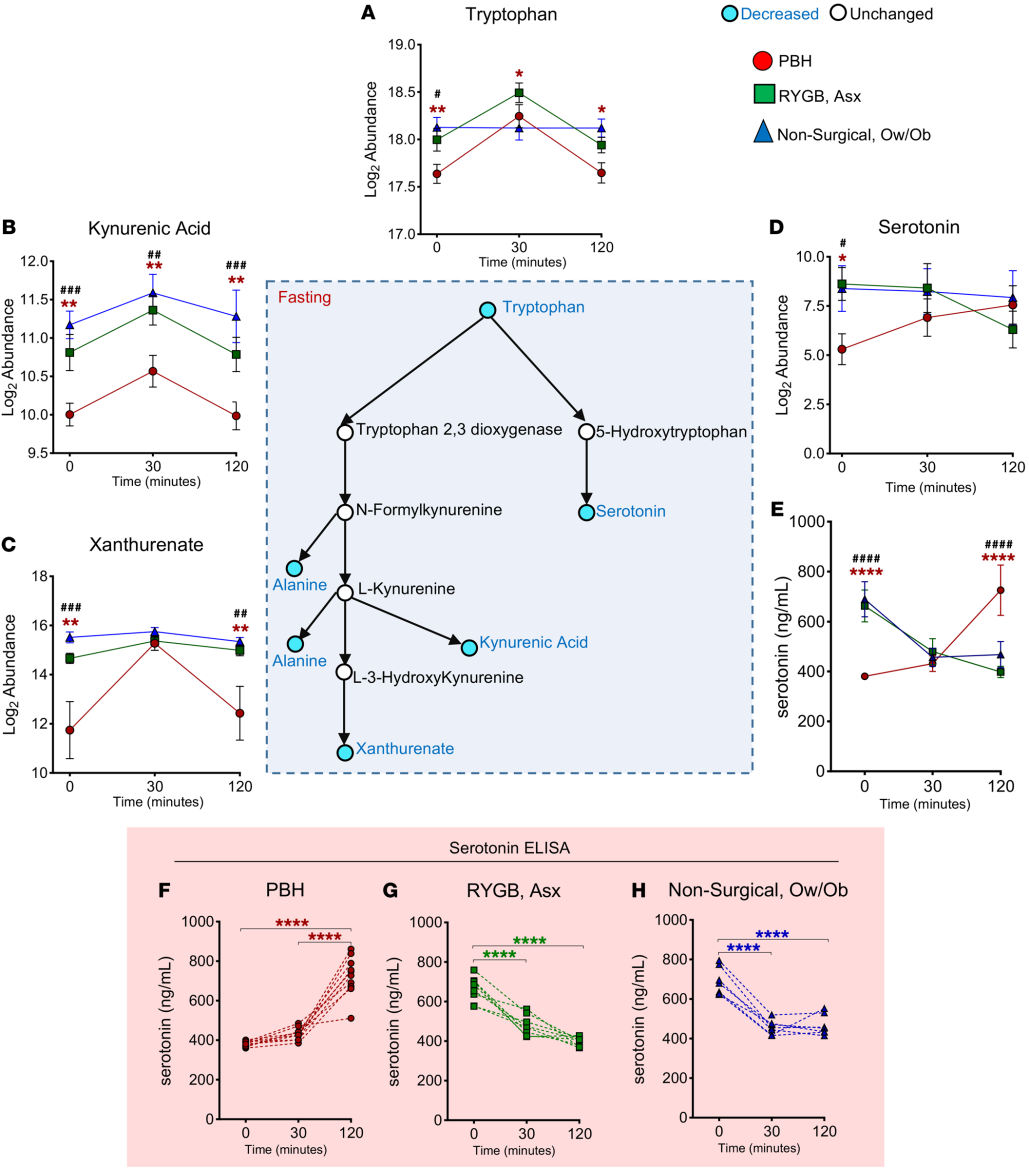
Abundance of tryptophan, tryptophan-derived metabolites, and serotonin in PBH, Asx, and Ow/Ob individuals in the fasting state and after mixed meal ingestion. Center: Schematic of tryptophan metabolism and downstream metabolites. (**A**–**E**) Abundance of tryptophan (**A**), kynurenic acid (**B**), xanthurenate (**C**), serotonin (**D**), and serotonin by ELISA (**E**). (**F**–**H**) Serotonin ELISA of individual participants in PBH (red), Asx (green), and Ow/Ob (blue) groups. Data are mean ± SD. *^,#^*P* < 0.05; **^,##^*P* < 0.01; ^###^*P* < 0.001;****P < 0.0001 for PBH vs. Asx; ^####^*P* < 0.0001 for PBH vs. Ow/Ob**.** Two-way ANOVA with Tukey’s multiple-comparison test was performed. Blue color of circles in the schematic indicates metabolites with decreased abundance, while white indicates unchanged abundance.

Both pathway and individual metabolite analysis revealed differential abundance of tryptophan pathway intermediates in PBH, including tryptophan itself and its downstream metabolites kynurenic acid, xanthurenate, and serotonin (Figure 4). For example, tryptophan levels were 40% lower in the fasting state in PBH versus Asx (*P* = 0.003, FDR = 0.06), and 20%–30% lower at 30 and 120 minutes (*P* = 0.028 and 0.019, respectively, FDR < 0.25) (Figure 4A). Similarly, kynurenic acid was reduced by 70% at all 3 time points (*P* < 0.01 for all, FDR < 0.25), while xanthurenate was 8-fold lower in the fasting state (*P* = 0.003, FDR = 0.06) and 6-fold lower at 2 hours (*P* = 0.008, FDR < 0.01) (Figure 4, B and C).

Serotonin levels were 10-fold lower in the fasting state in PBH, as compared with Asx (*P* = 0.019, FDR = 0.16) (Figure 4D). Interestingly, serotonin patterns in response to meal ingestion were distinct in PBH, increasing 3.2- and 4.7-fold at 30 and 120 minutes, respectively; by comparison, serotonin levels decreased after meal ingestion in Asx and nonsurgical groups.

To confirm alterations in serotonin dynamics in PBH, we analyzed serotonin using ELISA on samples collected in the fasting state and at 30 and 120 minutes after a meal originating from a newly recruited cohort described in Supplemental Table 5. Consistent with the metabolomics data, serotonin levels in individuals with PBH increased 1.9-fold after a meal (*P* < 0.001), whereas Asx and Ow/Ob patients had a 1.4- to 1.6-fold (*P* < 0.001 for both) decrease in serotonin levels over the same time course (Figure 4E). Serotonin levels for each participant are presented in Figure 4, F–H, demonstrating the distinct responses in all individuals with PBH.

Correlation analysis between metabolites and glucose levels during mixed meal tolerance test showed that tryptophan was correlated with glucose in the fasting state (*r* = 0.67, *P* < 0.001) and at 30 (*r* = 0.65, *P* < 0.001) and 120 minutes (*r* = 0.47, *P* = 0.023) (Supplemental Table 4). While there was no correlation between serotonin (measured by metabolomics assay) and glucose (Supplemental Table 4), plasma serotonin, measured by ELISA, was inversely correlated with glucose at 120 minutes after the meal in PBH (*r* = –0.78, *P* = 0.007) (Supplemental Figure 10).

To exclude the possibility that high plasma insulin or low glucose levels could contribute to observed increases in serotonin in the postprandial state, we analyzed serotonin levels in response to hyperinsulinemic-hypoglycemic clamps in PBH, Asx, and Ow/Ob participants (Supplemental Figure 16A). Consistent with the design of the clamp, plasma glucose levels at 100 minutes were similarly reduced in all groups (PBH: 51 ± 7 mg/dL; Asx: 51 ± 7 mg/dL; Ow/Ob: 53 ± 5 mg/dL; Supplemental Figure 16B). However, serotonin levels did not differ between groups, either in the fasting state (PBH: 103 ± 57 ng/mL; Asx: 152 ± 73 ng/mL; Ow/Ob: 174 ± 81 ng/mL) or during experimental hypoglycemia (PBH: 142 ± 72 ng/mL; Asx: 119 ± 89 ng/mL; Ow/Ob: 187 ± 53 ng/mL) (Supplemental Figure 16C). Similarly, serotonin levels did not change after insulin injection in mice (2 U/kg, i.p.) (Supplemental Figure 16, D and E) as compared with equal-volume saline at either 15 minutes (insulin: 430 ± 166 ng/mL; vs. saline: 344 ± 56 ng/mL) or 30 minutes (insulin: 344 ± 87 ng/mL; vs. saline: 351 ± 175 ng/mL) (Supplemental Figure 16F). Together, these results in both humans and mice suggest that hyperinsulinemia or hypoglycemia per se is not likely to mediate increases in postprandial serotonin in PBH.

### Serotonin induces hypoglycemia in mice.

**Figure 5:**
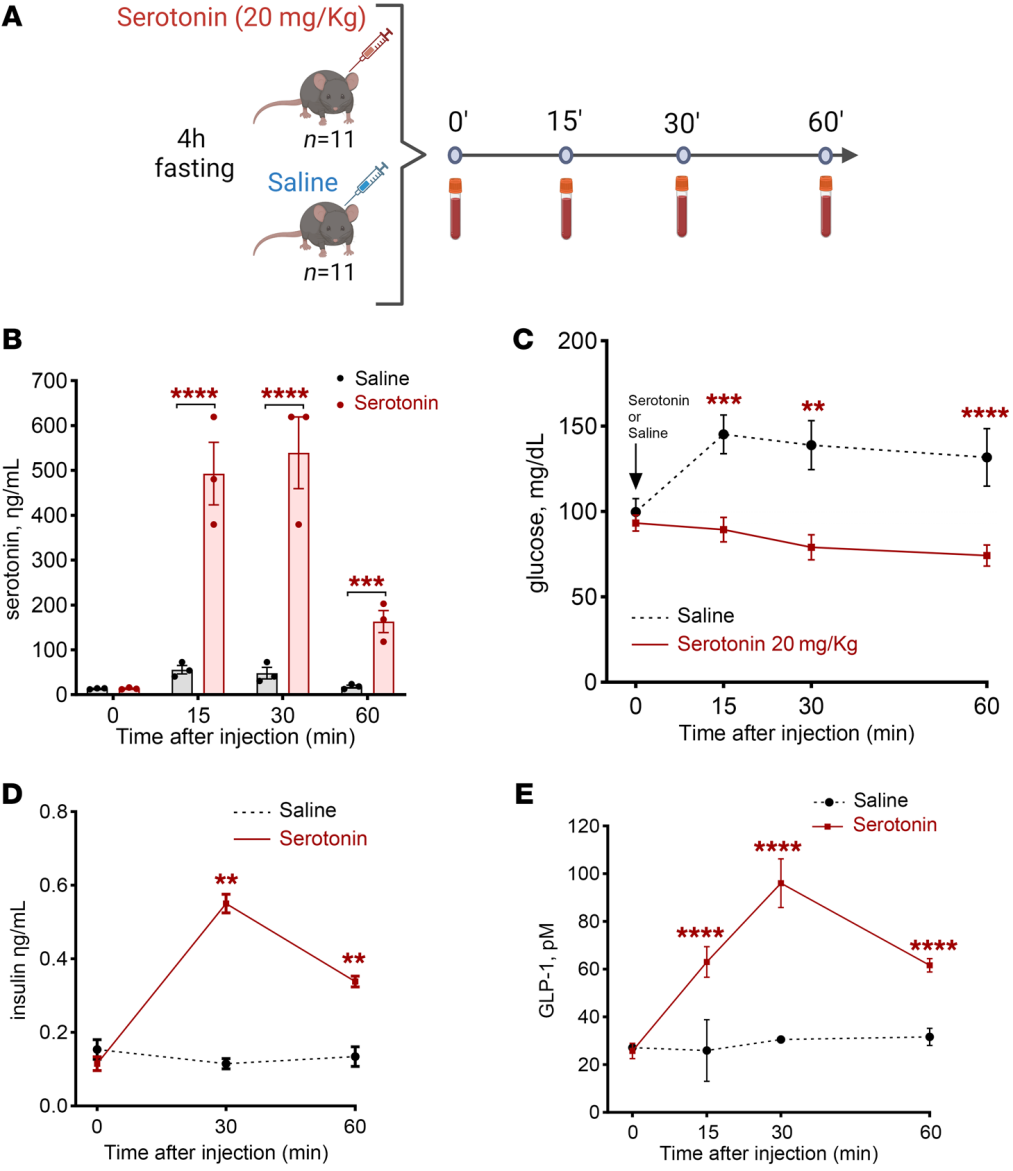
Serotonin reduces glucose and increases insulin and GLP-1 secretion. (**A**) Experimental protocol showing exogenous administration of serotonin (20 mg/kg) or saline in C57BL/6J mice. (**B**) Serotonin levels achieved by serotonin injection. (**C**) Glucose levels after serotonin or saline injection. (**D** and **E**) Insulin levels (**D**) and GLP-1 levels (**E**) after serotonin or saline injection. In all panels, **P < 0.01, ***P < 0.001, ****P < 0.0001 by 2-way ANOVA with Tukey’s multiple-comparison test.

Given that serotonin levels were uniquely increased in the postprandial state in individuals with PBH (4.7-fold at 120 minutes), and not influenced by experimental hypoglycemia or hyperinsulinemia, we sought to determine whether serotonin could modulate glucose levels in vivo in healthy C57BL/6J mice. We injected wild-type mice with serotonin (20 mg/kg, i.p.) or an equal volume of saline (Figure 5A). In serotonin-injected mice, serotonin levels increased rapidly to a peak of 539 ng/mL at 30 minutes (Figure 5B). To determine the impact of serotonin on glucose metabolism, we measured blood glucose at baseline and at 15, 30, and 60 minutes. Glucose levels were significantly reduced in serotonin-injected male mice at 15 (–39%, *P* < 0.001), 30 (–43%, *P* < 0.002), and 60 minutes (–44%, *P* < 0.018) (Figure 5C), with reductions of similar magnitude in females (–29% at 15 minutes, *P* < 0.001; –48% at 30 minutes, *P* < 0.001; and –48% at 60 minutes, *P* = 0.005) (Supplemental Figure 11). We next assessed whether serotonin-induced reduction in glucose was mediated by an insulin-dependent mechanism; plasma insulin increased 5- and 2.5-fold at 30 and 60 minutes after serotonin injection versus saline (*P* = 0.004 and *P* = 0.002) (Figure 5D). Interestingly, serotonin administration also significantly increased plasma GLP-1 levels 1.6- to 2.1-fold at 15, 30, and 60 minutes after injection (*P* = 0.011, *P* < 0.001, and *P* < 0.001, respectively) (Figure 5E).

**Figure 6:**
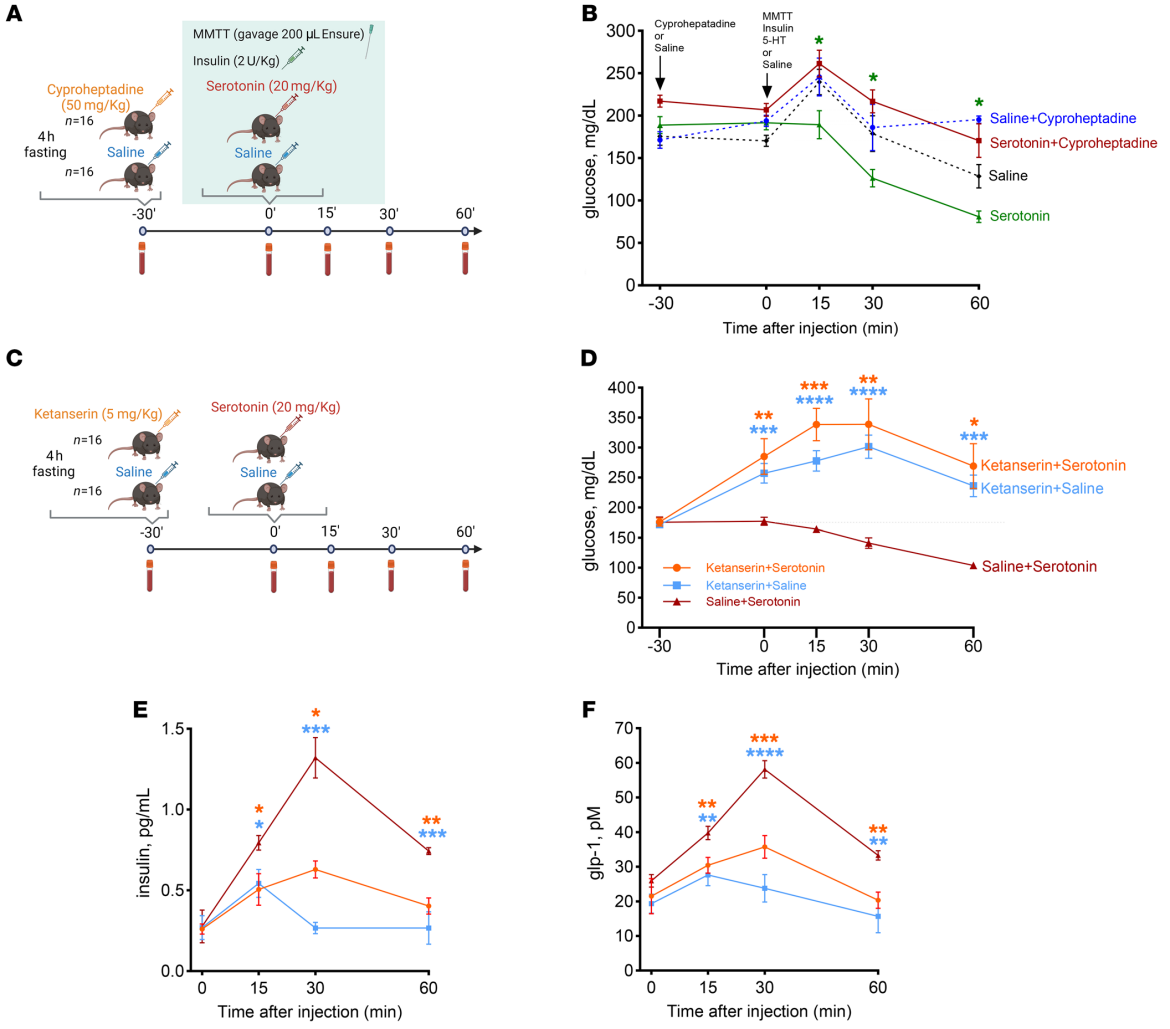
Serotonin induces hypoglycemia during meal tolerance test; hypoglycemia is blocked by cyprophetadine and more specifically by ketanserin. Reductions in glucose are blocked by the serotonin antagonist cyproheptadine and the specific serotonin receptor 2 antagonist ketanserin. (**A**) Scheme showing administration of cyproheptadine (50 mg/kg) or saline and, after 30 minutes, oral gavage with 200 μL of Ensure, insulin injection (2 U/kg), and administration of serotonin (20 mg/kg) or saline in C57BL/6J mice. MMTT, mixed meal tolerance test. (**B**) Glucose levels during experiment. (**C**) Scheme showing administration of ketanserin (5 mg/kg) and exogenous administration of serotonin (20 mg/kg) or saline in C57BL/6J mice. (**D**) Glucose levels after serotonin or saline injection. (**E** and **F**) Insulin levels (**E**) and GLP-1 levels (**F**) after serotonin or saline injection. In all panels, *P < 0.05, **P < 0.01, ***P < 0.001, ****P < 0.0001 by 2-way ANOVA with Tukey’s multiple-comparison test.

To mimic the postprandial glucose dynamics and exaggerated insulin secretion characteristic of PBH in mice, we developed an insulin-augmented mixed meal tolerance test. After a 4-hour fast, mice were gavaged with a liquid mixed meal (200 μL Ensure Compact, Abbott Laboratories) and injected with insulin (2 U/kg, i.p.; Humulin R, Lilly), before injection with serotonin or saline (Figure 6A). Consistent with prior experiments, serotonin (20 mg/kg) also reduced glucose by 38% versus saline in this setting, achieving levels below fasting level (serotonin, 80 mg/dL, vs. saline, 128 mg/dL, at 60 minutes; *P* = 0.021; Figure 6B).

Given that serotonin increased GLP-1 levels, we asked whether GLP-1 receptor blockade would attenuate serotonin affects. We first confirmed that the GLP-1 receptor antagonist avexitide (30 mmol/kg) blocked semaglutide-induced increases in insulin secretion (Supplemental Figure 13B) and, in parallel, attenuated the hypoglycemic effects of semaglutide (Supplemental Figure 13C) and reduced GLP-1 levels (Supplemental Figure 13D). Next, we tested whether avexitide could block serotonin-induced hypoglycemia (Supplemental Figure 14A). Treatment of mice with avexitide rapidly increased glucose levels in comparison with saline-injected mice (*P* = 0.012 at 15 minutes). However, avexitide was unable to block serotonin-induced hypoglycemia (Supplemental Figure 14B). Likewise, avexitide did not affect serotonin-induced increases in insulin and GLP-1 (Supplemental Figure 14, C and D). Thus, these data suggest that GLP-1 receptor–mediated signaling is not likely to be a primary mechanism mediating serotonin-induced hypoglycemia.

### Serotonin-induced hypoglycemia in mice is blocked by serotonin receptor antagonism.

First, we sought to investigate pathways by which serotonin induced hypoglycemia. Serotonin has pleiotropic effects on many aspects of metabolism, mediated via multiple receptor subtypes (16).

To test whether the effects of serotonin on glucose levels could be blocked by broad antagonism of serotonin receptor subtypes, the nonspecific antagonist cyproheptadine or an equal volume of saline was injected intraperitoneally 30 minutes before the mixed meal (Figure 6A). Cyproheptadine completely blocked the hypoglycemia induced by serotonin administration, with glucose of 170 mg/dL at 60 minutes versus 80 mg/dL with serotonin alone (*P* = 0.005) (Figure 6B). Interestingly, cyproheptadine pretreatment also blunted the increase in plasma serotonin as compared with saline at 15 (75%, *P* < 0.001), 30 (72%, *P* = 0.008), and 60 minutes (74%, *P* = 0.034) (Supplemental Figure 12A). Likewise, serotonin-induced increase in GLP-1 (1.7- to 2.1-fold, *P* < 0.001; Supplemental Figure 12B) was abolished by pretreatment with cyproheptadine (–1.9- to –2.1-fold, *P* < 0.001 for all; Supplemental Figure 12B). These results suggest that increased plasma serotonin may contribute to regulation of postprandial glucose metabolism, potentially via effects to increase both GLP-1 and insulin secretion.

We next investigated whether inhibition of specific serotonin receptor subtypes could block serotonin-induced hypoglycemia, selecting inhibitors of 5-HTR_3_ and 5-HTR_2_, given their demonstrated role in metabolism and insulin secretion (17–19). The 5-HTR_3_ antagonist ondansetron (3 mg/kg) was administered 30 minutes before injection of serotonin (20 mg/kg) or an equal volume of saline (Supplemental Figure 15A). Ondansetron did not impact serotonin-induced reductions in glucose (Supplemental Figure 15B), nor was it able to block serotonin-stimulated increases in insulin (Supplemental Figure 15C) or GLP-1 (Supplemental Figure 15D).

We next tested the impact of the 5-HTR_2_ antagonist ketanserin, injecting male mice with ketanserin (5 mg/kg) 30 minutes before serotonin administration (20 mg/kg) (Figure 6C). Ketanserin abolished the impact of serotonin, increasing blood glucose by 86% (*P* < 0.05) (Figure 6D). Likewise, ketanserin reduced serotonin-stimulated insulin levels by 44% (*P* < 0.05) (Figure 6E) and reduced serotonin-stimulated GLP-1 levels by 33% (*P* < 0.05) (Figure 6F). These data suggest that 5-HTR_2_ may mediate glycemic effects of serotonin and that inhibition could be tested as a potential strategy for PBH.

## Discussion

PBH can be observed in up to 20%–30% of patients following bariatric surgery. When severe, it can be associated with neuroglycopenia, reduced awareness, and impaired safety. Accelerated gastric emptying with rapid increases in glucose and AA absorption, together with elevations in GLP-1 and other intestinally derived hormones, contributes to increased postprandial insulin secretion and subsequent hypoglycemia (20). Increased insulin-independent glucose uptake (4) and reduced counterregulatory responses (21) may also contribute to hypoglycemia in patients with PBH. Given that maintenance of normal glucose homeostasis also requires adequate substrate availability and metabolism, we performed and now report an unbiased metabolomics analysis of the plasma metabolome in the fasting state and in response to a standard mixed meal stimulus in individuals with PBH as compared with unaffected post-bariatric participants.

Our data suggest that metabolism in PBH is not simply an extreme example of post-bariatric metabolism, as multiple metabolites exhibited unique patterns in PBH, with differential direction of change as compared with asymptomatic post-RYGB individuals. For example, we observed PBH-specific increases in abundance of adenine, oxalate, and hydroxyphenylpyruvate. It is possible that unique differences in dietary patterns or intestinal or microbial metabolism could contribute to these PBH-specific metabolic responses. More detailed studies analyzing the intestinal microbiome in individuals with PBH will be required.

AA and lipid metabolism ultimately yields carbons for key metabolic pathways, such as oxidative TCA cycle activity and gluconeogenesis. Interestingly, several glycolytic intermediates were reduced in PBH in the fasting state, alongside an increase in the TCA cycle intermediates aconitate, isocitrate, and succinate. These patterns were largely normalized at the 30-minute time point but were observed again at 2 hours — patterns reminiscent of the accelerated onset of postprandial physiology after RYGB, potentially linked to rapid emptying of nutrients from the pouch (22), as well as accelerated return to a fasting state. Levels of the ketone β-hydroxybutyrate were reduced in response to the meal in all 3 groups, as expected. However, levels were significantly increased in PBH in both the fasting state and after 120 minutes, potentially reflecting increased fatty acid oxidation with fasting, an accelerated return to fasting physiology after meals (despite high postprandial insulin levels), and protective adaptation to provide alternative fuels in the setting of recurrent hypoglycemia (23).

Thus, we hypothesize that lipid metabolism is the dominant contributor to whole-body metabolism both in the fasting state and at 2 hours postprandially in PBH. In this setting, if gluconeogenic flux and/or glycogenolysis are also compromised as a result of inadequate substrates for gluconeogenesis (e.g., reduced AAs), subnormal glycogen storage (low caloric intake), or reduced counterregulatory hormones, hypoglycemia at late postprandial time points could ensue. The increase in 3-phosphoglycerate at 120 minutes suggests that reductions in gluconeogenesis at more distal steps could potentially contribute to hypoglycemia. While studies of flux will be required to dissect these interesting patterns, we hypothesize that PBH is characterized by enhanced lipid fuel utilization in the fasting state, but inadequate glucose fuel availability in the fed-fasted transition.

Individuals with PBH also had reductions in levels of B-complex vitamins such as niacinamide (B_3_) and pantothenate (B_5_), and levels of both B_3_ and B_5_ were correlated with fasting glucose. Reductions in these cofactors for critical enzymes could potentially impair the function of central metabolic pathways including the TCA cycle (24), and could suggest deficiencies in micronutrient intake or absorption in PBH.

Bile acids, now recognized as metabolically active signaling molecules (25), are increased after RYGB (26). While the sum of measured bile acids was not increased in PBH versus asymptomatic participants in this cohort, the conjugated bile acid taurocholate was 2.5-fold higher in PBH in the fasting state, consistent with prior reports (9, 11), and several species tended to be higher in post-surgical participants. Additional targeted studies of bile acid synthesis, conjugation, and microbial metabolism in the fasting and postprandial states will be required to further define these patterns.

Abundance of multiple AAs, including branched-chain AAs (BCAAs), was reduced in individuals with PBH as compared with both asymptomatic and nonsurgical groups in the fasting state. AAs increased rapidly after mixed meal ingestion in both surgical groups, potentially reflecting rapid gastric emptying and absorption. However, lower levels persisted in PBH over time, and total AA levels correlated with glucose levels both in the fasting state and at 30 minutes. These data are of interest for several reasons. Firstly, AAs have long been recognized as important substrates for gluconeogenesis and central metabolic pathways. For example, alanine, reduced in PBH, is a major substrate for hepatic gluconeogenesis (27). Multiple BCAAs were also reduced in PBH, including the ketogenic Leu, forming acetyl-CoA, the glucogenic Val, yielding succinyl-CoA, and isoleucine, which can be metabolized into both acetyl-CoA and succinyl-CoA. Secondly, our data are concordant with prior reports showing reductions in AAs, BCAAs, and downstream products of BCAA catabolism following RYGB (22, 28, 29). Given that elevations in AAs are associated with obesity and insulin resistance (30) and predict risk of T2D (31), the further reduction in AAs in patients with PBH as compared with unaffected post-surgical patients may reflect the impact of weight loss, altered nutrition, and improved insulin sensitivity to yield an exaggerated “anti-diabetes” metabolic state contributing to reductions in gluconeogenesis. Alterations in AAs in PBH could also contribute to the significant reduction in 2-aminoadipate and α-hydroxybutyrate, biomarkers of diabetes risk and insulin resistance (12, 13, 32).

We do not fully understand the mechanisms underlying reductions in multiple AAs in PBH. Given that differences were observed mainly for essential AAs, it will be important to determine whether sources or quantity of dietary protein intake, absorption (33), or metabolism differs in post-surgical individuals with and without PBH. The rapid prandial excursions in AAs after RYGB could also contribute to prandial rises in insulin and glucagon (34, 35). Additional studies of in vivo flux will be required to clarify these possibilities.

A key finding is that multiple metabolites within the tryptophan pathway are downregulated in the fasting state in PBH as compared with asymptomatic individuals, including tryptophan and its downstream metabolites kynurenic acid and xanthurenic acid. Tryptophan was also significantly correlated with glucose at all 3 time points. It is possible that altered intake and/or microbial metabolism of tryptophan could contribute to reduced absorption and reduced plasma levels of tryptophan and downstream metabolites after bariatric surgery (36, 37).

Even more striking were differences in levels of serotonin in individuals with PBH, which were validated by ELISA in samples from an independent validation cohort. Firstly, fasting serotonin levels were 10-fold lower in PBH, as compared with both unaffected post-surgical individuals and nonsurgical participants. Secondly, responses to the mixed meal were distinct and divergent in PBH. Consistent with prior reports in healthy individuals (38), serotonin levels decreased after meal ingestion in both Asx and nonsurgical groups. By contrast, patients with PBH had a 5-fold *increase* in meal-stimulated serotonin.

Several lines of evidence suggest that observed changes in serotonin in the postprandial state may contribute mechanistically to postprandial increases in GLP-1 and insulin in PBH. Firstly, our studies in both humans and mice indicate that neither experimental hyperinsulinemia nor hypoglycemia results in increased serotonin. Secondly, we demonstrate that experimental injection of serotonin can potently reduce blood glucose in mice, consistent with prior studies (39, 40), in parallel with greater than 2-fold increases in insulin and GLP-1. We also extended these observations to the postprandial state; serotonin injection at the time of oral mixed meal gavage also induced hypoglycemia.

Unique patterns of alterations in serotonin levels in both the fasting and the postprandial state are of great interest for the pathophysiology of PBH given its role in multiple key processes in both peripheral tissues and the central nervous system linked to maintenance of normal glucose homeostasis (41–45). Serotonin modulates insulin secretion (46, 47) and contributes to expansion of β cell mass during pregnancy. Furthermore, production and release of serotonin by β cells (48) inhibits glucagon secretion from α cells (46). Serotonin also impacts intestinal metabolism, including stimulation of GLP-1 secretion by intestinal L cells (49, 50) and modulation of the local enteric and central nervous system (51). Central serotoninergic signaling also regulates food intake; loss of the 5-HT_2C_ receptor leads to obesity. Moreover, serotoninergic neurons are required for glucagon secretion, a key element of the counterregulatory response to hypoglycemia (52).

Clinically, modulation of serotonin has been linked to hypoglycemia. Antidepressant selective serotonin reuptake inhibitors (SSRIs) or serotonin-norepinephrine reuptake inhibitors (SNRIs) and monoamine oxidase (MAO) inhibitors, which inhibit metabolism of serotonin, can cause hypoglycemia. Moreover, we recently reported that use of SSRIs and SNRIs was a significant risk factor for self-reported hypoglycemia symptoms in post-RYGB patients enrolled in the Longitudinal Assessment of Bariatric Surgery (LABS) cohort (53). Interestingly, symptoms experienced by PBH patients in the postprandial state show some similarities to those in individuals who have serotonin syndrome, a condition induced by SSRIs and other serotoninergic medications (54) and manifested by confusion, sweating, tremors, and, in more severe cases, seizures (55). However, there were no differences in medication classes, including SSRIs and SNRIs, in any of the cohorts used for the current studies (Supplemental Table 5).

Taken together, our data support the hypothesis that dysregulation of serotonin metabolism, with reduction in fasting levels and increased meal-stimulated levels, may contribute to excessive prandial insulin secretion and hypoglycemia in patients with PBH. We do not yet understand the mechanisms responsible for the reduction in basal serotonin levels and the unique increases in postprandial serotonin levels in individuals with PBH. Potential mediators of increased postprandial serotonin levels in PBH could include increased numbers of serotonin-secreting enterochromaffin cells, or increased release of serotonin by these same cells, potentially linked to alterations in luminal contents (56). Conversely, altered expression or activity of serotonin synthesis enzymes or cofactors (e.g., vitamin B_6_) and serotonin-metabolizing enzymes (e.g., MAO) could also result in increased postprandial levels. Indeed Ben-Zvi and colleagues (57) demonstrated that expression of MAO is reduced 2- to 5-fold in jejunum and ileum after bariatric surgery in mice. In turn, increased serotonin could contribute to increases in meal-stimulated GLP-1 and insulin secretion characteristic of PBH, as we now demonstrate in mice. It is possible that these interactions are potentiated by PBH-specific alterations in gut microbiome and/or bile acid signaling or turnover (58). This concept is supported by several studies demonstrating that the intestinal microbiome plays a critical role in serotonin metabolism, bile acid production, and even intestinal motility (59–61). Whether differences in serotonin are preexisting prior to surgery in those who develop PBH, or solely develop in response to the altered intestinal anatomy postoperatively, is an interesting question for future studies.

Our findings suggest opportunities for targeting serotonin metabolism as a potential approach to the management of PBH. Our pilot experiments using the nonspecific serotonin receptor antagonist cyproheptadine reduced serotonin-induced effects on glucose. While previous studies identified the serotonin 5-HT_3_ receptor in β cells as a mediator of serotonin-induced insulin secretion (47, 62), here we demonstrate that serotonin receptor 3 antagonist (ondansetron) was not able to block serotonin-induced hypoglycemia. By contrast, administration of serotonin receptor 2 antagonist (ketanserin) abolished the impact of serotonin to induce hypoglycemia, in concert with decreased insulin and GLP-1 secretion. These data suggest that serotonin receptor subtype 2 might mediate serotonin effects on hypoglycemia, potentially via impact on pancreatic β cells and intestinal L cells (18, 19, 56). Future studies will be required to test whether serotonin receptor blockade could be an effective strategy for PBH therapy, and to ascertain whether additional serotonin receptor subtypes also contribute in diverse tissues and cell types relevant to PBH, such as L cells, enterocytes, enterochromaffin cells, islets, liver, and brain (56, 63).

We acknowledge several limitations of our study. Firstly, the mass spectrometry methodology used to analyze metabolomics is semiquantitative, permitting only relative between-group analysis. However, differences in serotonin between groups were confirmed by ELISA. Secondly, the sample size is relatively small, with a majority of women, which does not allow generalization to other populations. In mouse studies, we demonstrate that serotonin-mediated reduction in glucose is similar in both males and females. Thirdly, given the observational nature of our study, it is not possible to determine whether observed changes in levels of metabolites reflect changes in dietary patterns, nutrient absorption, catabolism, or flux. Because the postoperative period was between 8 and 12 years, we do not have a record of current or previous nutritional intake of supplements such as B-complex vitamins. Fourthly, while the intestine is the dominant source of circulating serotonin, future studies will be required to determine whether intestinally derived serotonin is the mediator of metabolic and hormonal changes characteristic of PBH and whether neural signaling is required. Finally, we acknowledge that the diet, the gut microbiome, and its interactions with the host may contribute to differences in metabolite profiles in both the fasting and postprandial states between patients with PBH and asymptomatic and nonsurgical controls (64).

In summary, we identify metabolic signatures of PBH including reduction in circulating AAs and tryptophan intermediates, multiple lipid species, and increased TCA cycle intermediates. These potentially contribute to alterations in glucose metabolic flux in both the fasting and the postprandial state, which collectively increase risk for hypoglycemia. We also identified PBH-specific patterns of serotonin dynamics in both the fasting and the postprandial state. Using a mouse model, we demonstrate that exogenous serotonin administration in mice lowers plasma glucose, potentially via increases in both plasma insulin and GLP-1 levels. Moreover, serotonin-induced hypoglycemia after a mixed meal was blocked by the serotonin antagonist cyproheptadine and more specifically by the serotonin receptor 2 antagonist ketanserin. Thus, increases in postprandial serotonin levels may contribute to postprandial symptoms and reduction in glucose levels, and provide a potential therapeutic target for PBH.

## Methods

### Sex as a biological variable.

In both cohorts of human participants, both male and female individuals were included. Sex was not considered as a biological variable, given the low percentage of males in our study population, consistent with the greater percentage of females among the post-surgical population, limiting power for sex-specific analysis. However, for animal studies, we evaluated both male and female mice; similar results suggest relevance for both sexes.

### Participants.

The Joslin Diabetes Center Institutional Review Board approved the study. Written informed consent was obtained from all participants. A detailed description of the previous clinical study that generated residual samples for analysis of the present study is provided in ref. 5. In brief, 3 study groups were analyzed (Supplemental Table 1): (a) PBH: 13 participants with history of RYGB and neuroglycopenia recruited from the hypoglycemia clinic at Joslin Diabetes Center; (b) asymptomatic post-RYGB group (Asx): 10 participants who underwent uncomplicated RYGB 2–4 years previously with no history of hypoglycemia; and (c) overweight/obese (Ow/Ob): 8 individuals without history of gastrointestinal surgery (control group). Blood samples were obtained after an overnight fast and at 30 and 120 minutes after consumption of a liquid mixed meal (Ensure, 9 g protein, 40 g carbohydrate, 6 g fat, 240 mL; Abbott Laboratories).

A new cohort (Supplemental Table 5) was recruited from 2020 to 2023 and included 15 participants with PBH, 15 Asx, and 10 Ow/Ob. Samples from this cohort were used to validate serotonin levels by ELISA during mixed meal tolerance test and during hyperinsulinemic-hypoglycemic clamps.

### Metabolomics analysis.

Plasma collected at time 0, 30, and 120 minutes after mixed meal (5, 9) was used for untargeted semiquantitative metabolomic analysis using flow injection–tandem mass spectrometry, with detection of 189 metabolites.

### Hyperinsulinemic-hypoglycemic clamp.

A primed, continuous infusion of insulin (80 mU/m^2^/min) was used together with a 20% glucose infusion, titrated every 5 minutes to gradually lower the glucose from fasting levels to a target of 50 mg/dL at a rate of 1 mg/dL/min (65); the target glucose was maintained for 30 minutes to ensure a consistent stimulus for counterregulatory responses.

### Animal studies.

All animal studies were approved by the Institutional Animal Care and Use Committee of Joslin Diabetes Center. C57BL/6J male and female mice, age 6 weeks, were purchased from The Jackson Laboratory. Mice were maintained on a 12-hour light/12-hour dark cycle with ad libitum access to water and chow diet.

The impact of serotonin was assessed after a 4-hour fast; mice were injected with 20 mg/kg of serotonin (serotonin hydrochloride, Sigma-Aldrich) or an equal volume of saline intraperitoneally. Blood was collected at baseline (before injection) and at 15, 30, and 60 minutes after injection from the tail vein or via cardiac puncture at the time of euthanasia in EDTA-coated microtubes containing DPP-IV inhibitor (Millipore); blood glucose was measured using the Prodigy AutoCode meter.

To mimic the postprandial glucose dynamics and high insulin secretion characteristic of PBH, the effect of serotonin on glucose metabolism in response to an insulin-augmented mixed meal tolerance test was assessed. After a 4-hour fast, mice were gavaged with a liquid mixed meal (200 μL Ensure Compact, Abbott Laboratories) and i.p. injection of insulin (2 U/kg; Humulin R, Lilly). Serotonin (20 mg/kg) or an equal volume of saline was injected i.p., immediately after insulin injection. For some experiments, mice were treated with the nonspecific serotonin antagonist cyproheptadine (50 mg/kg; MilliporeSigma) i.p. or an equal volume of saline, 30 minutes before mixed meal. For all experiments, blood glucose was measured in the fasting state, at the time of gavage (time 0), and at 15, 30, and 60 minutes thereafter.

In additional studies, mice were pretreated with the GLP-1 receptor antagonist avexitide (30 mmol/kg; Eiger Biopharmaceuticals), the serotonin receptor 3 antagonist ondansetron (3 mg/kg; Cayman Chemical), and the serotonin receptor 2 antagonist ketanserin (5 mg/kg; MilliporeSigma).

To determine whether insulin could impact serotonin levels in mice, mice fasted for 4 hours were injected i.p. with insulin (2 U/kg; Humulin R, Lilly) or saline. Blood was collected via tail vein at time 0 and 15 and 30 minutes after injection.

### ELISA and metabolite assays.

For human samples, plasma collected at baseline (fasting) and at 30 and 120 minutes after the mixed meal or plasma collected at baseline (fasting) and at 40 and 100 minutes after initiation of the hyperinsulinemic-hypoglycemic clamp was assayed by serotonin ELISA (ADI-900-175, Enzo) following the manufacturer’s instructions.

For mouse samples, plasma collected at fasting (time 0) and subsequently at 15, 30, and 60 minutes after injections was assayed by Ultra-Sensitive Mouse Insulin ELISA (90082, Crystal Chem), serotonin ELISA (ADI-900-175, Enzo), and mouse GLP-1 ELISA (81508, Crystal Chem). All sampled blood was collected via tail vein or cardiac puncture in EDTA-coated microtubes containing DPP-IV inhibitor (Millipore). All assays were performed according to manufacturers’ instructions.

### Statistics.

All metabolite data were log-transformed, and those with more than 75% missing values were filtered out. The remaining missing values were imputed with half of the minimum values of the corresponding metabolites. Each sample’s abundances were scaled so that all samples had the same median abundance. Differential abundance was assessed by linear regression modeling with limma, which applies an empirical Bayesian approach to moderate each metabolite’s variance (66). Two-group comparisons were done by moderated *t* tests and multiple-group comparisons by moderated *F* tests (both 2-tailed, unpaired). *P* values were corrected using the Benjamini-Hochberg false discovery rate (FDR) (67). Metabolite clusters in a hierarchical dendrogram were detected using a variable height cutoff approach (68). The volcano plot was made using ggplot2 (69) and heatmap by pheatmap (70). Metabolite pathway sets were downloaded from the Small Molecule Pathway Database (SMPDB) (71), and metabolite set enrichment was tested using the limma ROAST method (72). All bioinformatics analysis was done in the R software, and graphs were generated using R and GraphPad Prism 9 (GraphPad Software Inc.).

For non-metabolic data, data were analyzed using either 1- or 2-way ANOVA, as specified in the figure legends. The graphical abstract was created with BioRender (biorender.com).

### Study approval.

The study was approved by the Committee on Human Subjects, Joslin Diabetes Center (Boston, Massachusetts, USA). Written informed consent was obtained from all participants. Animal procedures were performed using protocols approved by the Institutional Animal Care and Use Committee of Joslin Diabetes Center.

### Data availability.

All data points presented in the graphs are detailed in the Supporting Data Values file.

## Author contributions

MEP conceived and designed the study. RFB, BO, CC, VE, PCQ, LP, HW, VN, HS, CMM, DS, HP, JMD, DCS, and MEP acquired, analyzed, or interpreted data. RFB and MEP drafted the manuscript. All authors critically revised the manuscript for important intellectual content. HP and JD performed statistical analysis. MEP obtained funding.

## Supplementary Material

Supplemental data

ICMJE disclosure forms

Supplemental table 2

Supplemental table 3

Supplemental table 4

Supporting data values

## Version 1 09/12/2024

In-Press Preview

## Version 2 11/01/2024

Electronic publication

## References

[1] Mingrone G (2021). Metabolic surgery versus conventional medical therapy in patients with type 2 diabetes: 10-year follow-up of an open-label, single-centre, randomised controlled trial. Lancet.

[2] Courcoulas AP (2024). Long-term outcomes of medical management vs bariatric surgery in type 2 diabetes. JAMA.

[3] Hansen CF (2013). Hypertrophy dependent doubling of L-cells in Roux-en-Y gastric bypass operated rats. PLoS One.

[4] Sandoval DA, Patti ME (2023). Glucose metabolism after bariatric surgery: implications for T2DM remission and hypoglycaemia. Nat Rev Endocrinol.

[5] Goldfine AB (2007). Patients with neuroglycopenia after gastric bypass surgery have exaggerated incretin and insulin secretory responses to a mixed meal. J Clin Endocrinol Metab.

[6] Tan M (2020). Safety, efficacy and pharmacokinetics of repeat subcutaneous dosing of avexitide (exendin 9-39) for treatment of post-bariatric hypoglycaemia. Diabetes Obes Metab.

[7] Lee D (2021). Glycemic patterns are distinct in post-bariatric hypoglycemia after gastric bypass (PBH-RYGB). J Clin Endocrinol Metab.

[8] Patti ME (2015). Insulin response to oral stimuli and glucose effectiveness increased in neuroglycopenia following gastric bypass. Obesity (Silver Spring).

[9] Mulla CM (2019). Plasma FGF-19 levels are increased in patients with post-bariatric hypoglycemia. Obes Surg.

[10] Kuhre RE (2018). Bile acids are important direct and indirect regulators of the secretion of appetite- and metabolism-regulating hormones from the gut and pancreas. Mol Metab.

[11] Patti ME (2009). Serum bile acids are higher in humans with prior gastric bypass: potential contribution to improved glucose and lipid metabolism. Obesity (Silver Spring).

[12] Gall WE (2010). α-Hydroxybutyrate is an early biomarker of insulin resistance and glucose intolerance in a nondiabetic population. PLoS One.

[13] Wang TJ (2013). 2-Aminoadipic acid is a biomarker for diabetes risk. J Clin Invest.

[14] Rhee EP (2011). Lipid profiling identifies a triacylglycerol signature of insulin resistance and improves diabetes prediction in humans. J Clin Invest.

[15] Plubell DL (2018). GM-CSF driven myeloid cells in adipose tissue link weight gain and insulin resistance via formation of 2-aminoadipate. Sci Rep.

[16] Yabut JM (2019). Emerging roles for serotonin in regulating metabolism: new implications for an ancient molecule. Endocr Rev.

[17] Engel M (2013). The serotonin 5-HT3 receptor: a novel neurodevelopmental target. Front Cell Neurosci.

[18] Bennet H (2016). Serotonin (5-HT) receptor 2b activation augments glucose-stimulated insulin secretion in human and mouse islets of Langerhans. Diabetologia.

[19] Nagata M (2019). Blockade of multiple monoamines receptors reduce insulin secretion from pancreatic β-cells. Sci Rep.

[20] Craig CM (2017). Critical role for GLP-1 in symptomatic post-bariatric hypoglycaemia. Diabetologia.

[21] Abrahamsson N (2016). Gastric bypass reduces symptoms and hormonal responses in hypoglycemia. Diabetes.

[22] Svane MS (2019). Postprandial nutrient handling and gastrointestinal hormone secretion after roux-en-Y gastric bypass vs sleeve gastrectomy. Gastroenterology.

[23] Newman JC, Verdin E (2014). Ketone bodies as signaling metabolites. Trends Endocrinol Metab.

[24] Kennedy DO (2016). B vitamins and the brain: mechanisms, dose and efficacy—a review. Nutrients.

[25] Houten SM (2006). Endocrine functions of bile acids. EMBO J.

[26] Sachdev S (2016). FGF 19 and bile acids increase following Roux-en-Y gastric bypass but not after medical management in patients with type 2 diabetes. Obes Surg.

[27] Felig P (1970). Alanine: key role in gluconeogenesis. Science.

[28] Laferrere B (2011). Differential metabolic impact of gastric bypass surgery versus dietary intervention in obese diabetic subjects despite identical weight loss. Sci Transl Med.

[29] Dreyfuss JM (2021). High-throughput mediation analysis of human proteome and metabolome identifies mediators of post-bariatric surgical diabetes control. Nat Commun.

[30] Newgard CB (2009). A branched-chain amino acid-related metabolic signature that differentiates obese and lean humans and contributes to insulin resistance. Cell Metab.

[31] Pereira S (2008). Insulin resistance of protein metabolism in type 2 diabetes. Diabetes.

[32] Landaas S (1975). The formation of 2-hydroxybutyric acid in experimental animals. Clin Chim Acta.

[33] Bojsen-Moller KN (2015). Accelerated protein digestion and amino acid absorption after Roux-en-Y gastric bypass. Am J Clin Nutr.

[34] Schmid R (1989). Role of amino acids in stimulation of postprandial insulin, glucagon, and pancreatic polypeptide in humans. Pancreas.

[35] Wang X (2015). Multiple factors related to the secretion of glucagon-like peptide-1. Int J Endocrinol.

[36] O’Mahony SM (2015). Serotonin, tryptophan metabolism and the brain-gut-microbiome axis. Behav Brain Res.

[37] Martin CR (2018). The brain-gut-microbiome axis. Cell Mol Gastroenterol Hepatol.

[38] Ho JE (2013). Metabolite profiles during oral glucose challenge. Diabetes.

[39] Yamada J (1989). Serotonin-induced hypoglycemia and increased serum insulin levels in mice. Life Sci.

[40] Furman BL (1974). The hypoglycaemic effect of 5-hydroxytryptophan. Br J Pharmacol.

[41] D’Agostino G (2018). Brown adipose tissue controls skeletal muscle function via the secretion of myostatin. Cell Metab.

[42] Heisler LK (2006). Serotonin reciprocally regulates melanocortin neurons to modulate food intake. Neuron.

[43] Hanley NR, Van de Kar LD (2003). Serotonin and the neuroendocrine regulation of the hypothalamic—pituitary-adrenal axis in health and disease. Vitam Horm.

[44] Puglisi-Allegra S, Andolina D (2015). Serotonin and stress coping. Behav Brain Res.

[45] Sandi C, Haller J (2015). Stress and the social brain: behavioural effects and neurobiological mechanisms. Nat Rev Neurosci.

[46] Almaca J (2016). Human beta cells produce and release serotonin to inhibit glucagon secretion from alpha cells. Cell Rep.

[47] Kim K (2015). Functional role of serotonin in insulin secretion in a diet-induced insulin-resistant state. Endocrinology.

[48] Rorsman P, Ashcroft FM (2018). Pancreatic β-cell electrical activity and insulin secretion: of mice and men. Physiol Rev.

[49] Lund ML (2020). L-cell differentiation is induced by bile acids through GPBAR1 and paracrine GLP-1 and serotonin signaling. Diabetes.

[50] Ripken D (2016). Nutrient-induced glucagon like peptide-1 release is modulated by serotonin. J Nutr Biochem.

[51] Jenkins TA (2016). Influence of tryptophan and serotonin on mood and cognition with a possible role of the gut-brain axis. Nutrients.

[52] Martin H (2023). Serotonergic neurons are involved in the counter-regulatory response to hypoglycemia. J Neuroendocrinol.

[53] Fischer LE (2021). Postbariatric hypoglycemia: symptom patterns and associated risk factors in the Longitudinal Assessment of Bariatric Surgery study. Surg Obes Relat Dis.

[54] Francescangeli J (2019). The serotonin syndrome: from molecular mechanisms to clinical practice. Int J Mol Sci.

[55] Dunkley EJ (2003). The Hunter Serotonin Toxicity Criteria: simple and accurate diagnostic decision rules for serotonin toxicity. QJM.

[56] Lund ML (2018). Enterochromaffin 5-HT cells – A major target for GLP-1 and gut microbial metabolites. Mol Metab.

[57] Ben-Zvi D (2018). Time-dependent molecular responses differ between gastric bypass and dieting but are conserved across species. Cell Metab.

[58] Watanabe H (2010). Peripheral serotonin enhances lipid metabolism by accelerating bile acid turnover. Endocrinology.

[59] Ge X (2017). Antibiotics-induced depletion of mice microbiota induces changes in host serotonin biosynthesis and intestinal motility. J Transl Med.

[60] Ge X (2018). Intestinal crosstalk between microbiota and serotonin and its impact on gut motility. Curr Pharm Biotechnol.

[61] Ramirez-Perez O (2017). The role of the gut microbiota in bile acid metabolism. Ann Hepatol.

[62] Ohara-Imaizumi M (2013). Serotonin regulates glucose-stimulated insulin secretion from pancreatic β cells during pregnancy. Proc Natl Acad Sci U S A.

[63] Choi W (2018). Serotonin signals through a gut-liver axis to regulate hepatic steatosis. Nat Commun.

[64] Levy M (2015). Microbiota-modulated metabolites shape the intestinal microenvironment by regulating NLRP6 inflammasome signaling. Cell.

[65] Lembo E (2018). Implementation of low glycemic index diet together with cornstarch in post-gastric bypass hypoglycemia: two case reports. Nutrients.

[66] Ritchie ME (2015). limma powers differential expression analyses for RNA-sequencing and microarray studies. Nucleic Acids Res.

[67] Y Benjamini, Y Hochberg (1995). Controlling the false discovery rate: a practical and powerful approach to multiple testing. J R Stat Soc Ser B Methodol.

[68] Langfelder P (2008). Defining clusters from a hierarchical cluster tree: the Dynamic Tree Cut package for R. Bioinformatics.

[70] https://cran.r-project.org/web/packages/pheatmap/index.html.

[71] Jewison T (2014). SMPDB 2.0: big improvements to the Small Molecule Pathway Database. Nucleic Acids Res.

[72] Wu D (2010). ROAST: rotation gene set tests for complex microarray experiments. Bioinformatics.

