# Supplementary material for: Postprandial metabolomics analysis reveals disordered serotonin metabolism in post-bariatric hypoglycemia
Source: J Clin Invest. 2024 Sep 12;134(21):e180157. doi: 10.1172/JCI180157 (PMC11527454; doi:10.1172/JCI180157)
Supplement: Supplemental data [file jci-134-180157-s177.pdf]

LIST OF SUPPLEMENTARY MATERIALS for Ferraz-Bannitz R et al.

Supplementary Fig. 1. Metabolic and hormonal response to mixed meal tolerance test in the fasting state, 30 minutes and 120 minutes after mixed meal ingestion.

Supplementary Fig. 2. Metabolites demonstrating opposite directions of abundance compared between (Asx vs Ow/Ob) and (PBH vs Asx) at time 0 (fasting) (A), 30 minutes (B), and 120 minutes (C) after meal ingestion.

Supplementary Fig. 3. Pathway enrichment analysis of the down-regulated (blue) and up-regulated (red) metabolites in the comparison of PBH with Overweight/Obese without history of gastrointestinal surgery groups.

Supplementary Fig. 4. Pathway enrichment analysis of the down-regulated (blue) and up-regulated (red) metabolites in the comparison of asymptomatic post-RYGB with Overweight/Obese without history of gastrointestinal surgery groups.

Supplementary Fig. 5. Heatmap showing the correlation for the 50 top-ranking metabolites for which fasting abundance correlates with glucose levels at 120 minutes after mixed meal ingestion.

Supplementary Fig. 6. Abundance of lipid species, ketone and bile acid in PBH, Asx and Ow/Ob individuals at fasting state, 30 minutes and 120 minutes after mixed meal ingestion.

Supplementary Fig. 7. Abundance of biomarkers of diabetes and insulin resistance risk and B-complex vitamins in PBH, Asx and Ow/Ob individuals at fasting state, 30 minutes and 120 minutes after mixed meal ingestion.

Supplementary Fig. 8. Abundance of amino acids, per group and per participant in PBH, Asx and Ow/Ob groups at fasting state, 30 minutes and 120 minutes after mixed meal ingestion.

Supplementary Fig. 9. Correlation between glucose levels and amino acids in the fasting state and 30 minutes after mixed meal ingestion in individuals with PBH.

Supplementary Fig. 10. Correlation between glucose levels and serotonin in the fasting state (A), and at 30 (B) and 120 minutes (C) after mixed meal ingestion in PBH (red), Asx (green) and Ow/Ob (blue) individuals.

Supplementary Fig. 11. Serotonin induces hypoglycemia in female mice.

Supplementary Figure 12. Serotonin induces hypoglycemia and is blocked by the serotonin antagonist cyproheptadine.

Supplementary Figure 13. GLP-1 antagonist (avexitide) blocks the effects of GLP-1 agonist (semaglutide).

Supplementary Figure 14. Administration of GLP-1 antagonist (avexitide) does not block serotonin-induced hypoglycemia.

Supplementary Figure 15. Serotonin induces hypoglycemia and is not blocked by the serotonin antagonist receptor 3 ondansetron.

Supplementary Fig. 16. Glucose and serotonin levels during hyperinsulinemic hypoglycemic clamp in PBH, Asx and Ow/Ob individuals and insulin tolerance test using high dose insulin in mice.

Supplementary Table 1. Clinical characteristics of participants.

Supplementary Table 2. ID list of clusters

Supplementary Table 3. Full statistical analysis

Supplementary Table 4. Metabolites correlated with glucose levels at fasting state, 30 and 120 minutes after meal ingestion

Supplementary Table 5. Clinical characteristics of cohort participants for serotonin ELISA analysis during mixed meal and hyperinsulinemic hypoglycemic clamp.

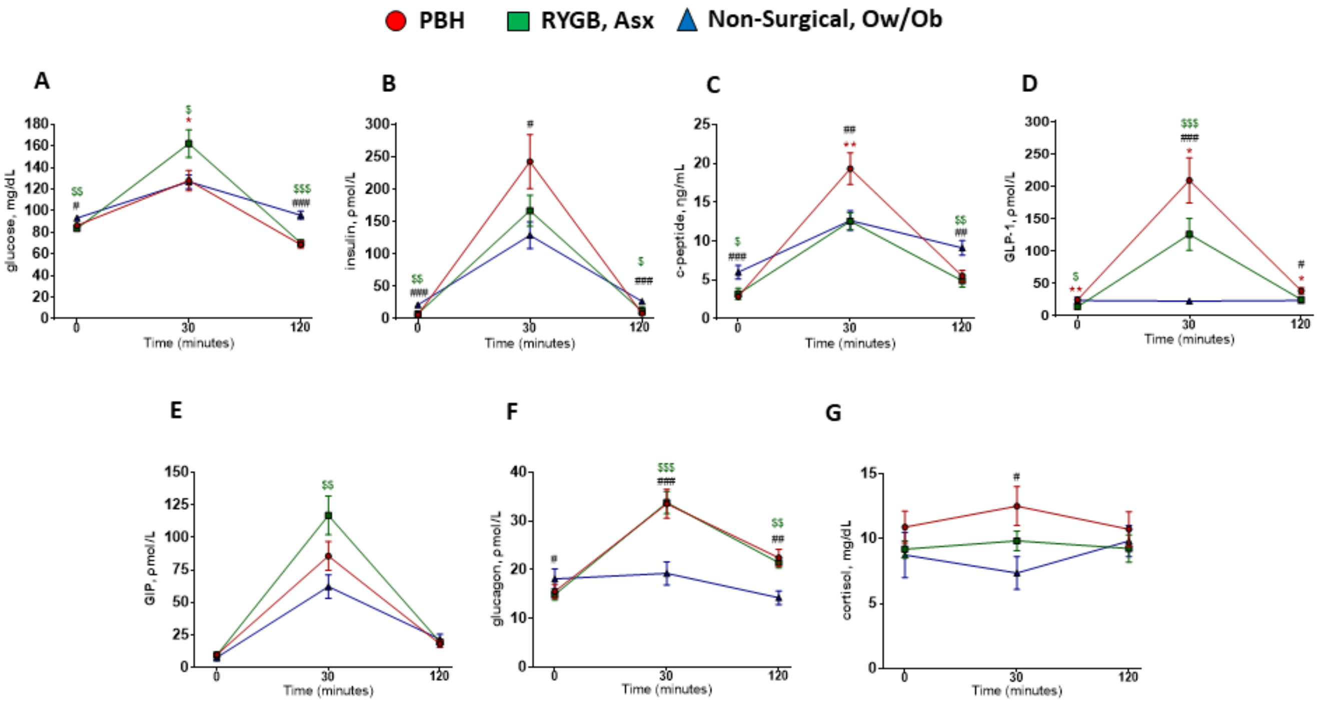

Supplementary Fig. 1. Metabolic and hormonal response to mixed meal tolerance test in the fasting state, 30 minutes and 120 minutes after mixed meal ingestion. Plasma concentrations of (A) Glucose, (B) Insulin, (C) C-peptide, (D) GLP-1 (E) GIP, (F) Glucagon, (G) Cortisol. Data are mean ± SD. *PBH vs Asx . #PBH vs Ow/Ob. $Asx vs Ow/Ob. *, #, $, $P < 0.05$; **, ##, $$, $P < 0.01$; ***, ###, $$$, $P < 0.001$. PBH (post-bariatric hypoglycemia), Asx (asymptomatic post-RYGB) and Ow/Ob (Overweight/obese without history of gastrointestinal surgery). ANOVA 2-way with Tukey’s multiple comparisons test was performed.

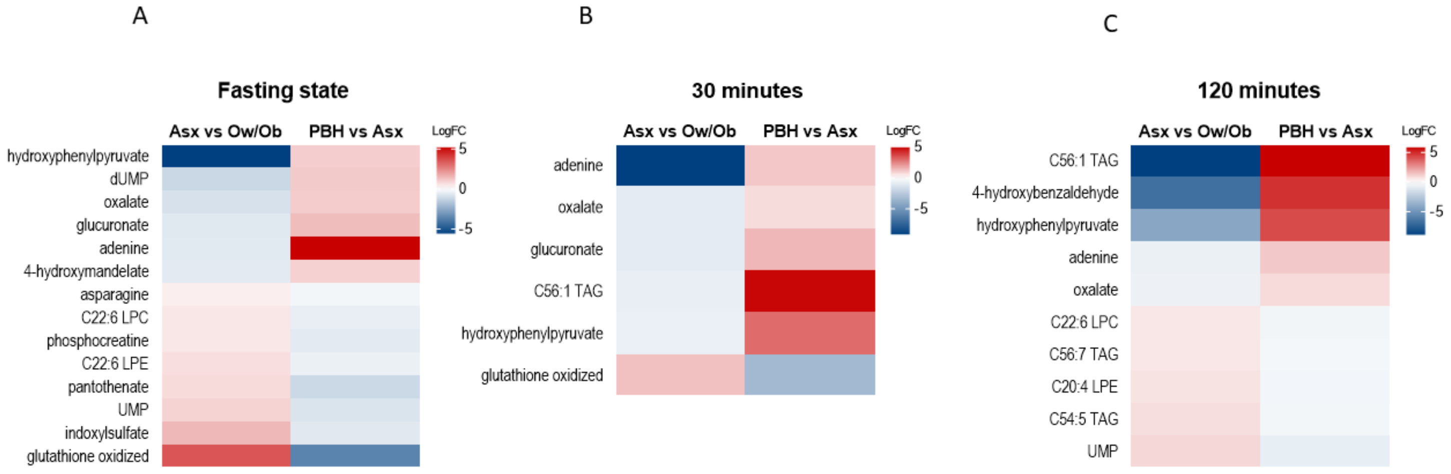

Supplementary Fig. 2. Metabolites demonstrating opposite directions of abundance compared between (Asx vs Ow/Ob) and (PBH vs Asx) at time 0 (fasting) (A), 30 minutes (B), and 120 minutes (C) after meal ingestion. Fold changes are log transformed and indicated by color scale in matrix. All metabolites represented in this figure had $P < 0.05$ for the indicated comparison.

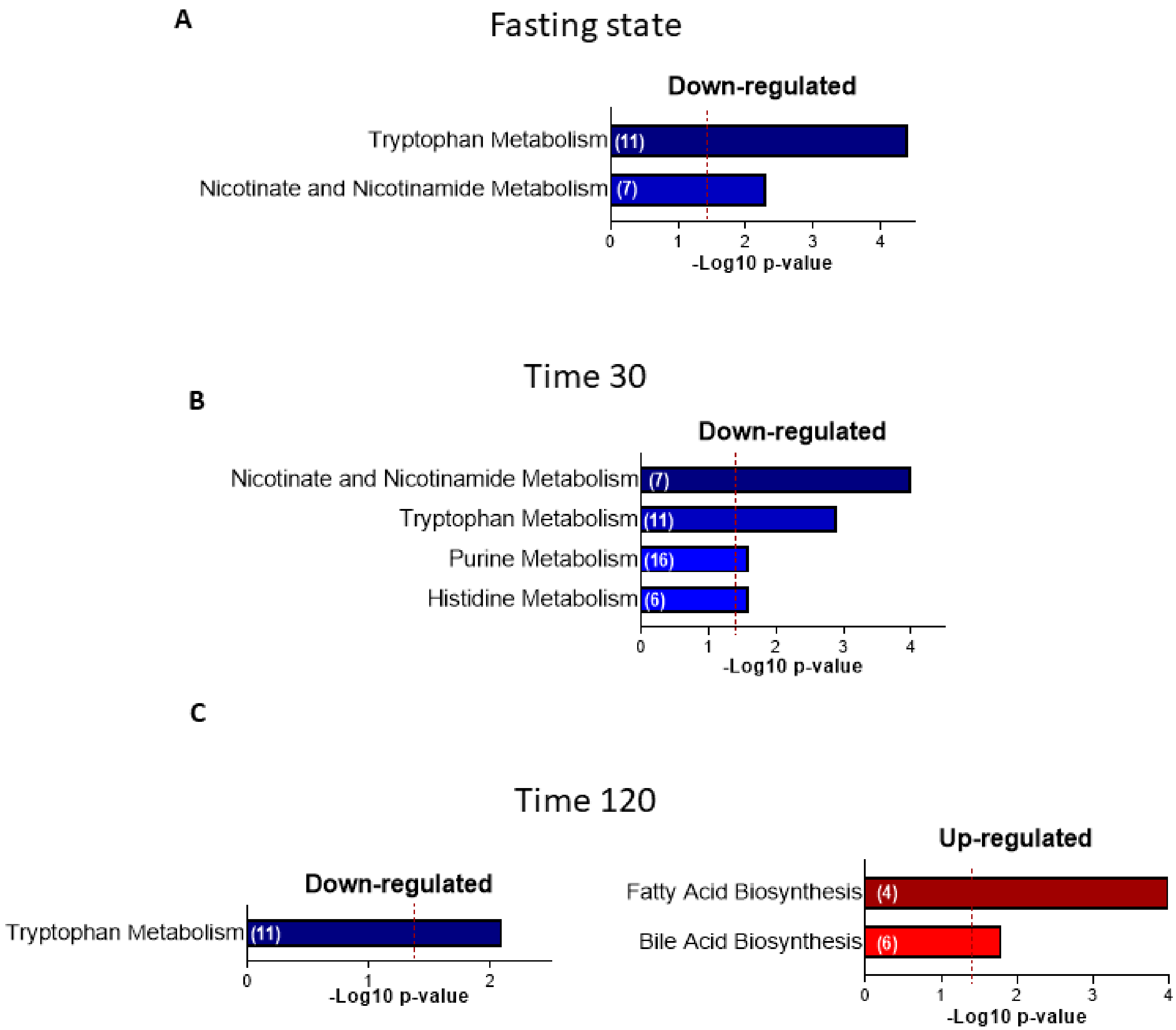

Supplementary Fig. 3. Pathway enrichment analysis of the down-regulated (blue) and up-regulated (red) metabolites in the comparison of PBH vs. Overweight/Obese (without history of gastrointestinal surgery). (A) Pathway enrichment in the fasting state, (B) 30 minutes and (C) 120 minutes after mixed meal ingestion. The $-\log_{10}$ p value for enrichment is represented on the x axis; the vertical line indicate nominal $p<0.05$.

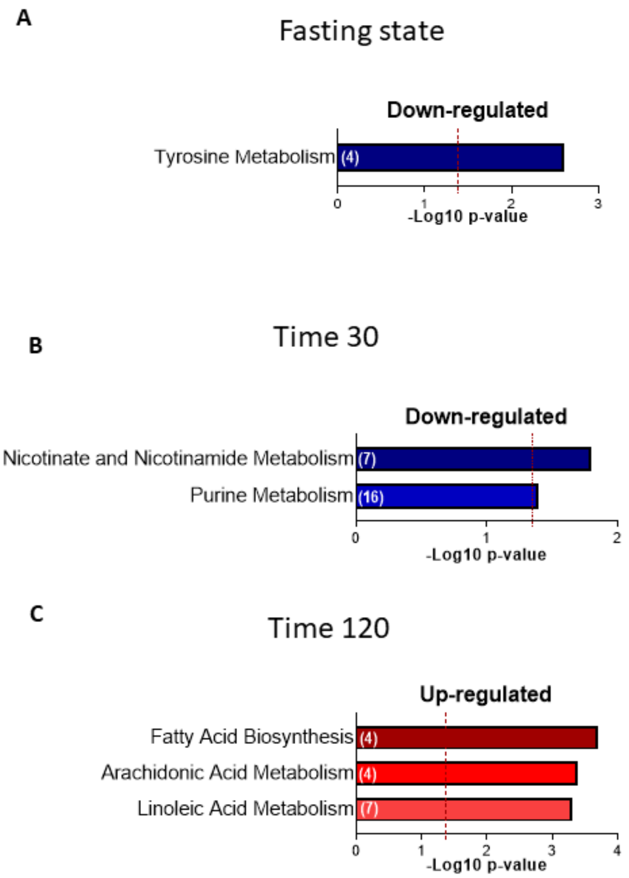

Supplementary Fig. 4. Pathway enrichment analysis of the down-regulated (blue) and up-regulated (red) metabolites in the comparison of asymptomatic post-RYGB vs. Overweight/Obese (without history of gastrointestinal surgery). (A) Pathway enrichment in the fasting state, (B) 30 minutes and (C) 120 minutes after mixed meal ingestion. The $-\log_{10}$ p value for enrichment is represented on the x axis; the vertical line indicates nominal $p<0.05$.

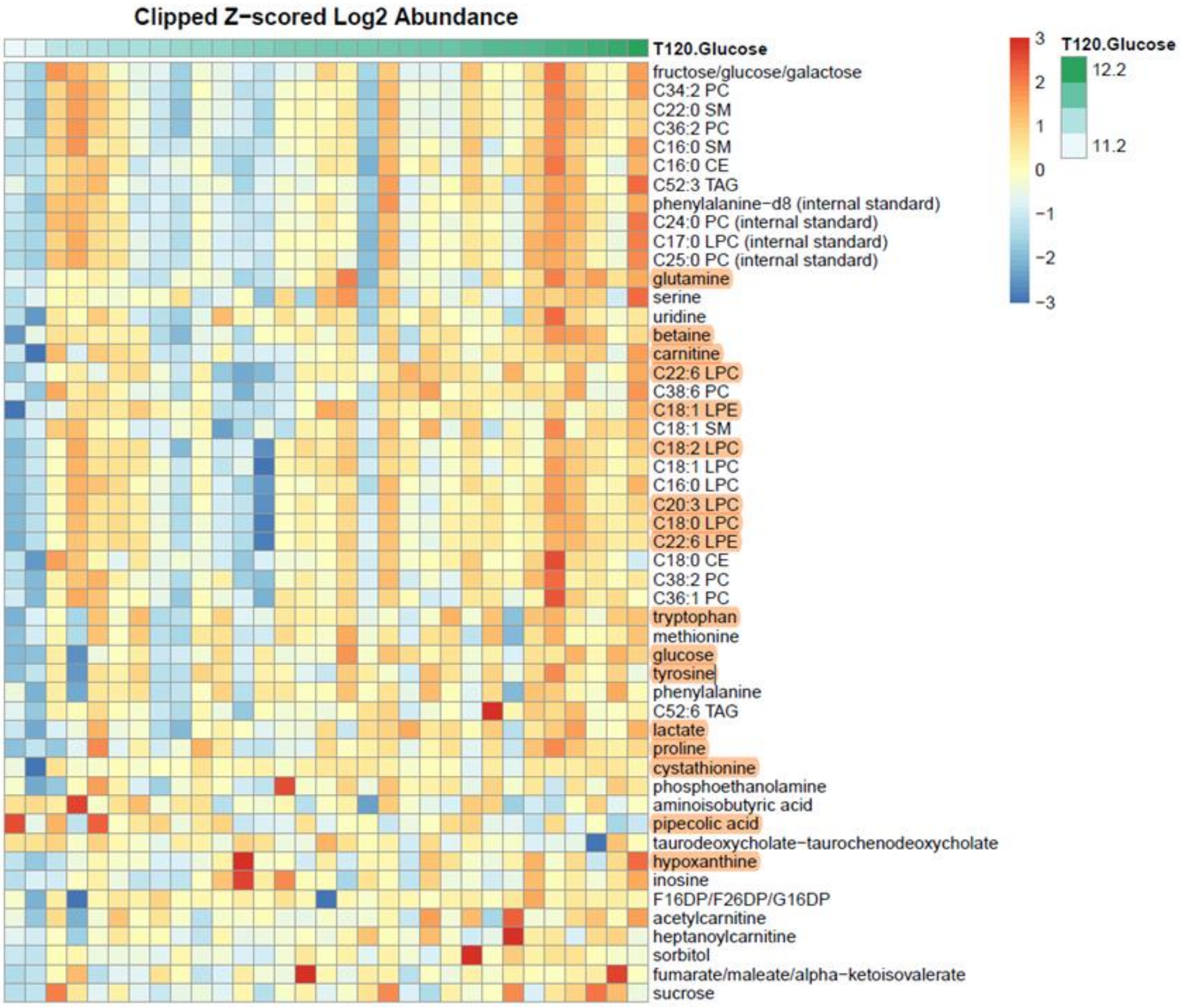

Supplementary Fig. 5. Heatmap showing the abundance of the 50 top-ranking metabolites for which fasting abundance correlates with glucose levels at 120 minutes after mixed meal ingestion. Metabolites indicated in orange represent FDR <0.25 for correlation.

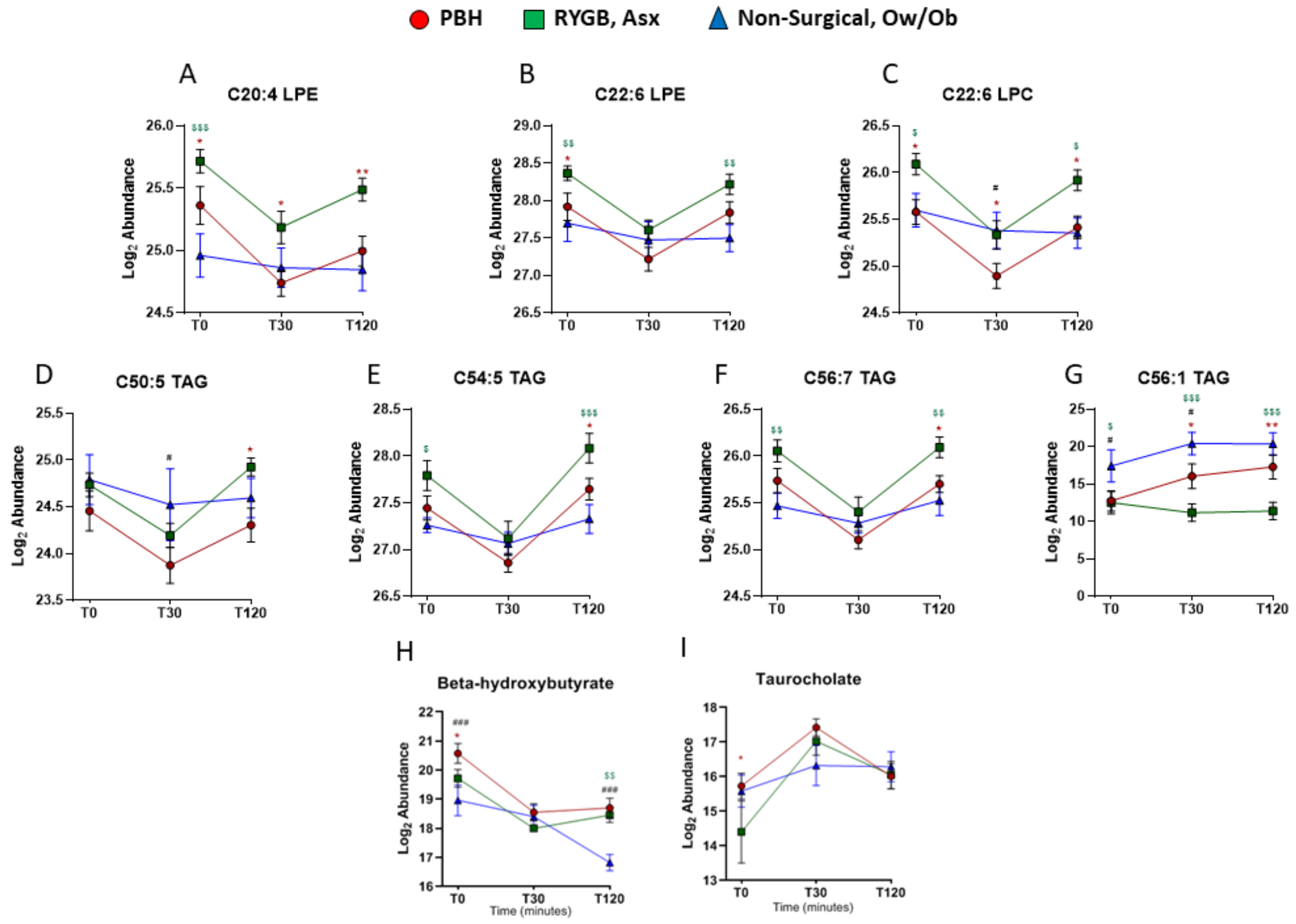

Supplementary Fig. 6. Abundance of lipid species, ketone and bile acid in PBH, Asx and Ow/Ob individuals in fasting state and at 30 minutes and 120 minutes after mixed meal ingestion. (A) Abundance of C20:4 LPE, (B) C22:6 LPE, (C) C22:6 LPC, (D) C50:5 TAG, (E) C54:5 TAG, (F) C56:7 TAG, (G) C56:1 TAG, (H) β-hydroxybutyrate (ketone), (I) Taurocholate (bile acid). Data are mean ± SD. *, #, $, $P < 0.05$; **, ##, $$, $P < 0.01$; ***, ###, $$$, $P < 0.001$. PBH (post-bariatric hypoglycemia), Asx (asymptomatic post-RYGB) and Ow/Ob (Overweight/obese without history of gastrointestinal surgery). ANOVA 2-way with Tukey's multiple comparisons test was performed.

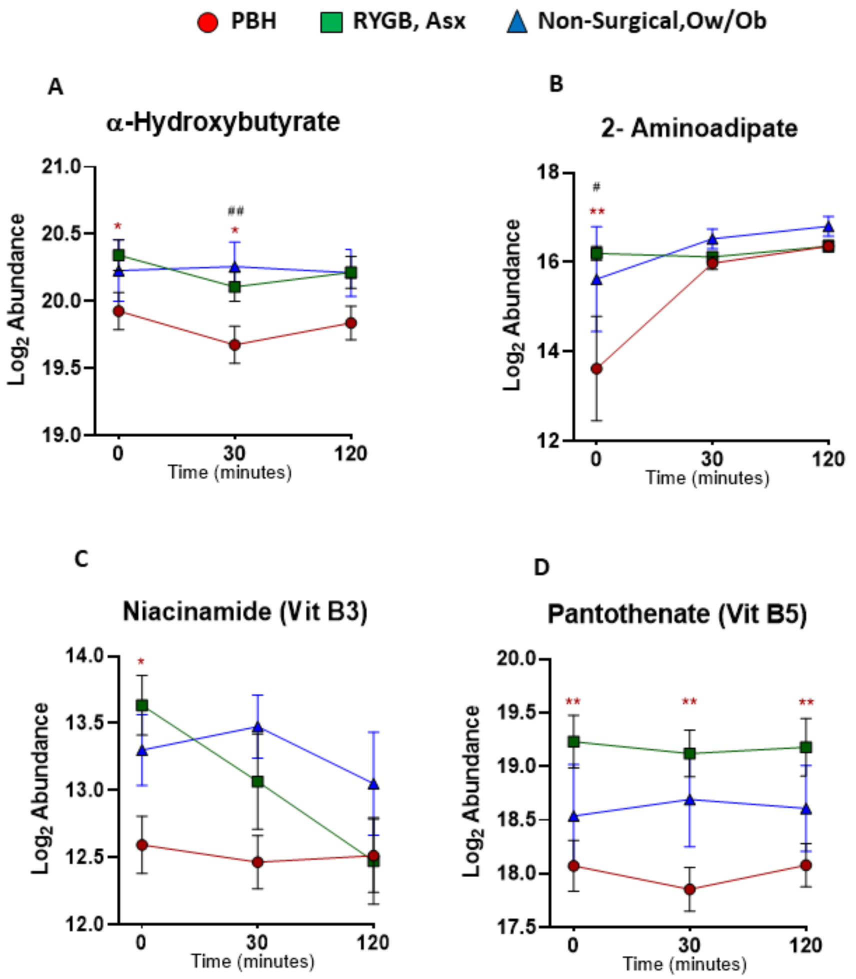

Supplementary Fig. 7. Abundance of biomarkers of diabetes and insulin resistance risk and B-complex vitamins in PBH, Asx and Ow/Ob individuals in fasting state and at 30 minutes and 120 minutes after mixed meal ingestion. (A) Alpha-Hydroxybutyrate, (B) 2-Aminoadipate, (C) Niacinamide (Vitamin B3), (D) Pantothenate (Vitamin B5). Data are mean ± SD. *, #, $, $P < 0.05$; **, ##, $$, $P < 0.01$; ***, ###, $$$, $P<0.001$. PBH (post-bariatric hypoglycemia), Asx (asymptomatic post-RYGB) and Ow/Ob (Overweight/obese without history of gastrointestinal surgery). ANOVA 2-way with Tukey's multiple comparisons test was performed.

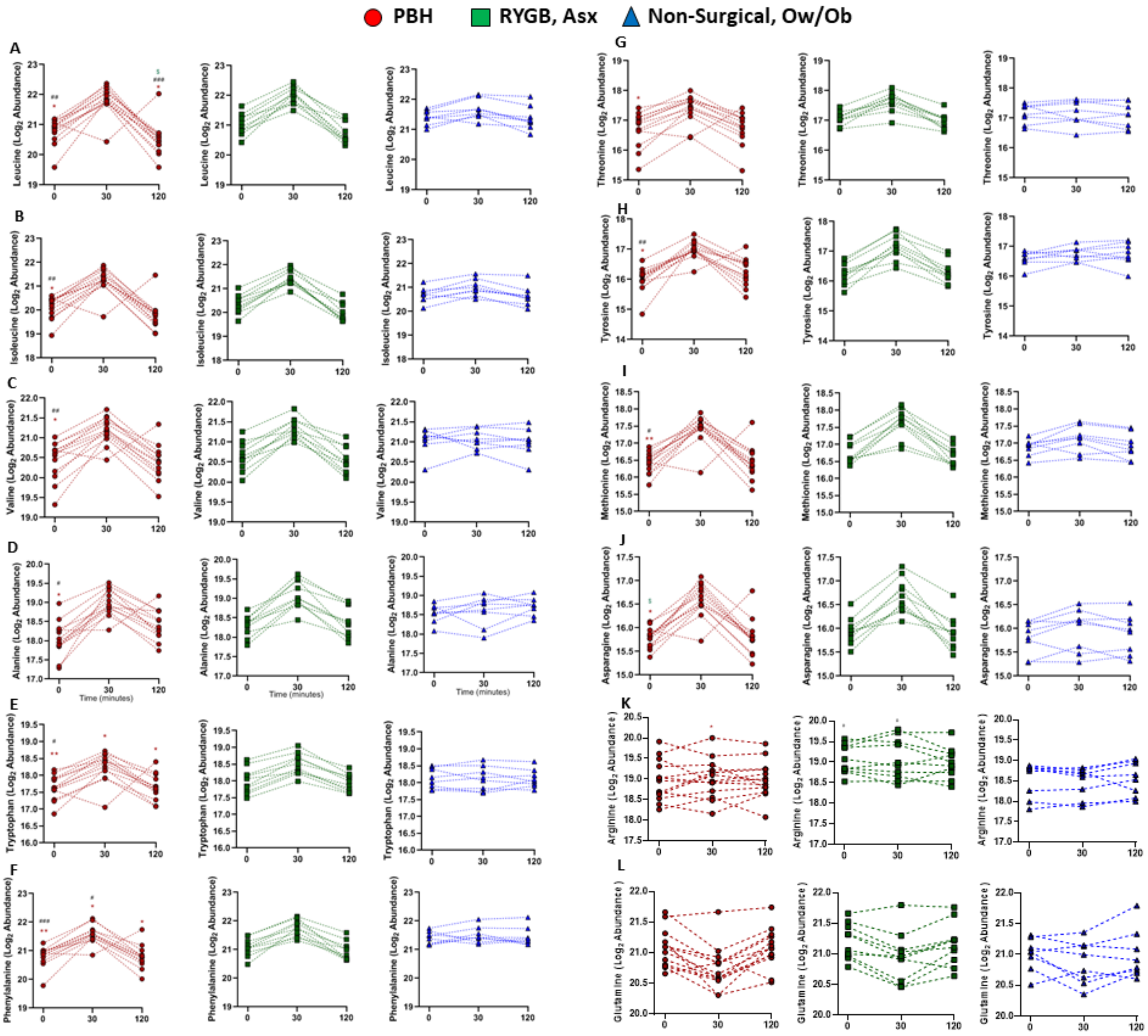

Supplementary Fig. 8. Abundance of amino acids, per group and per participant in PBH, Asx and Ow/Ob groups at fasting state, 30 minutes and 120 minutes after mixed meal ingestion. (A) Abundance of Leucine, (B) Isoleucine, (C) Valine, (D) Alanine, (E) Tryptophan, (F) Phenylalanine, (G) Threonine, (H) Tyrosine, (I) Methionine, (J) Asparagine, (K) Arginine and (L) Glutamine. Data are mean ± SD. *, #, $, P < 0.05; **, ##, $$, P < 0.01; ***, ###, $$$, P < 0.001. PBH (post-bariatric hypoglycemia), Asx (asymptomatic post-RYGB) and Ow/Ob (Overweight/obese without history of gastrointestinal surgery). ANOVA 2-way with Tukey's multiple comparisons test was performed.

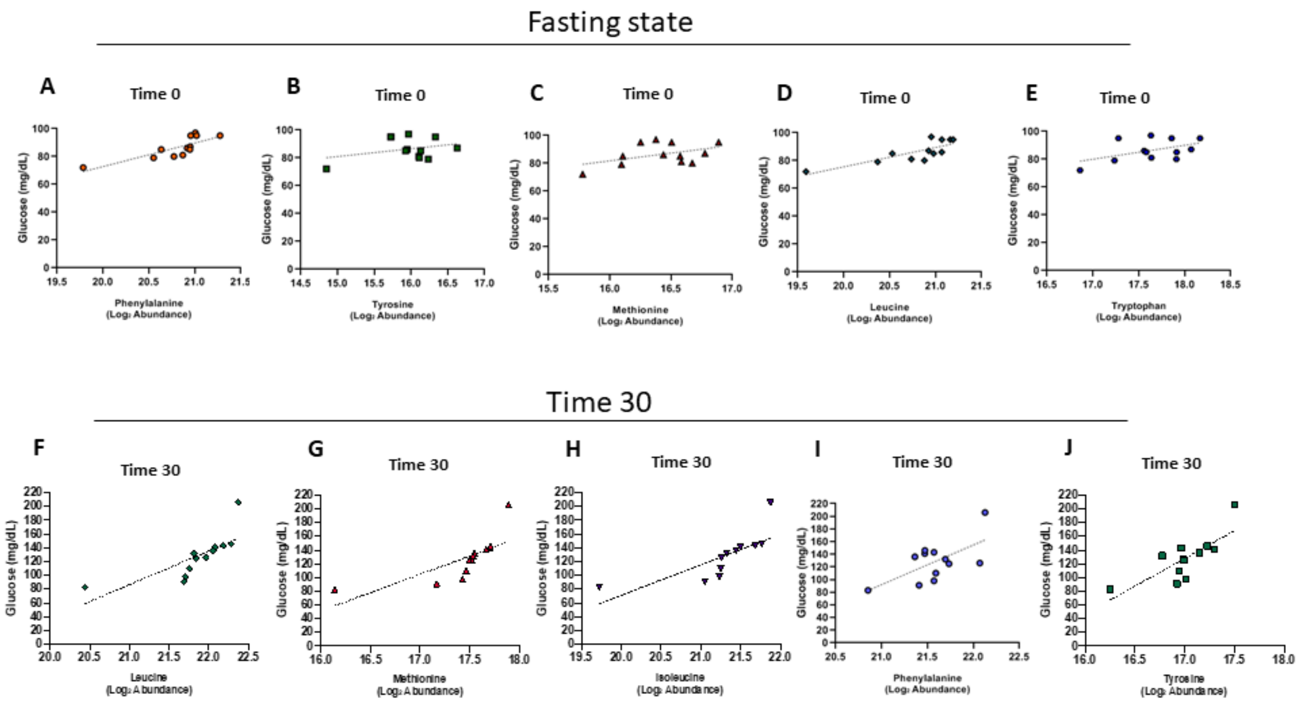

Supplementary Fig. 9. Correlation between glucose levels and amino acids in individuals with PBH. Fasting state: (A) Phenylalanine (r=0.76), (B) Tyrosine (r=0.74), (C) Methionine (r=0.72), (D) Leucine (r=.69), (E) Tryptophan (r=0.67). **Postprandial state (30 minutes after mixed meal):** (F) Leucine (r=0.77), (G) Methionine (r=0.76), (H) Isoleucine (r=0.76), (I) Phenylalanine (0.76), (J) Tyrosine (r=0.75). Statistical evaluations were performed using Pearson's rank correlation test.

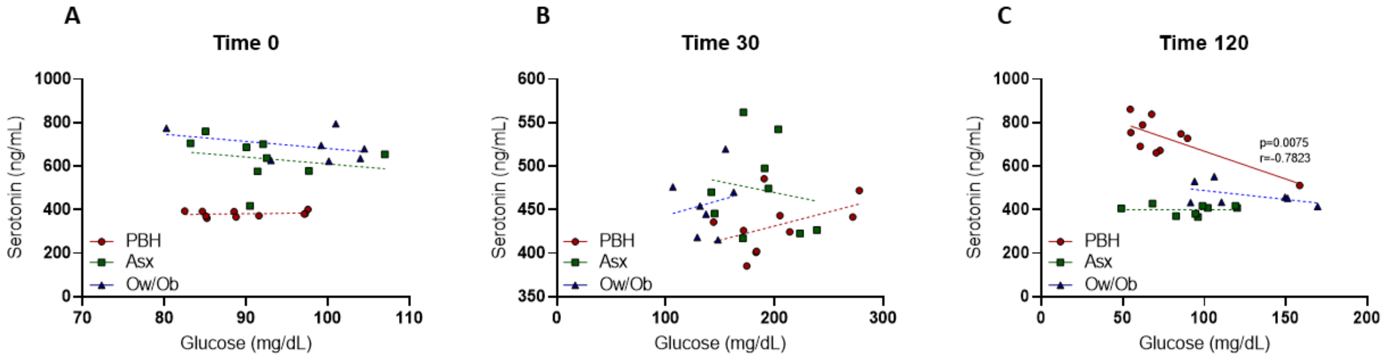

Supplementary Fig. 10. Correlation between glucose levels and serotonin in the fasting state (A), and at 30 (B) and 120 minutes (C) after mixed meal ingestion in PBH (red), Asx (green) and Ow/Ob (blue) individuals. Statistical evaluations were performed using Pearson's rank correlation test. * r=0.78, p<0.01 for correlation between serotonin and glucose at 120 minutes. PBH (red), Asx (green) and Ow/Ob (blue).

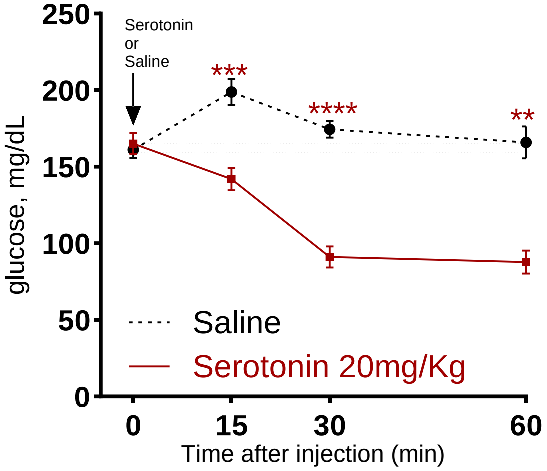

Supplementary Fig. 11. Serotonin reduces glucose in female mice. Glucose levels after serotonin (20 mg/kg) or saline injection. **$p<0.01$, ***$p<0.001$, ****$p<0.0001$ by ANOVA 2-way with Tukey's multiple comparisons test.

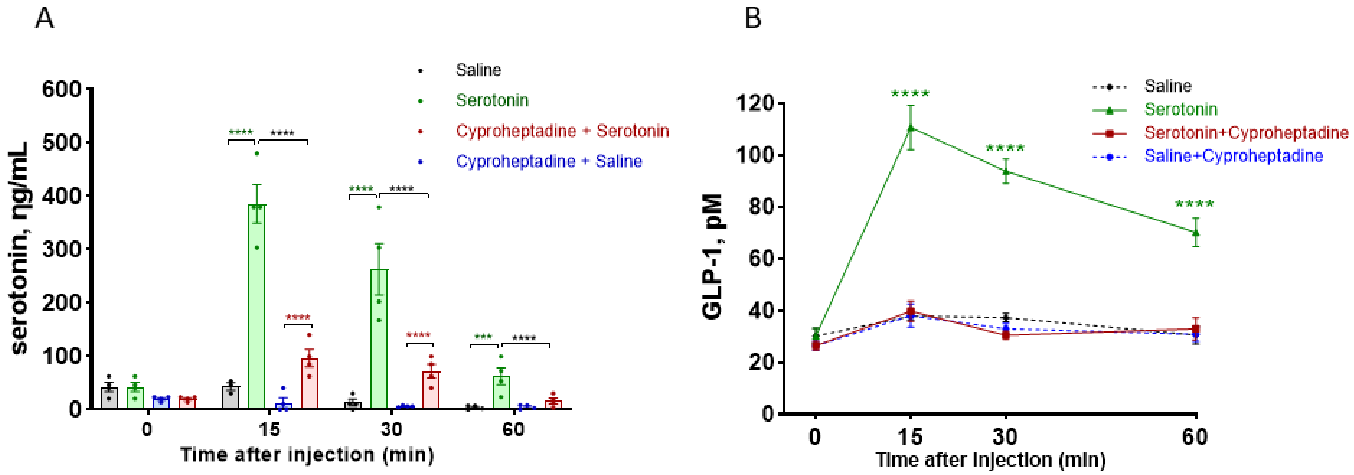

Supplementary Figure 12. Impact of the serotonin receptor antagonist cyproheptadine on (A) serotonin levels and (B) GLP-1 levels at fasting state, 15 min, 30 min and 60 min after concomitant oral gavage, insulin injection and administration of serotonin or saline. In all panels, **$p<0.01$, ***$p<0.001$, ****$p<0.0001$ by ANOVA 2-way with Tukey's multiple comparisons test.

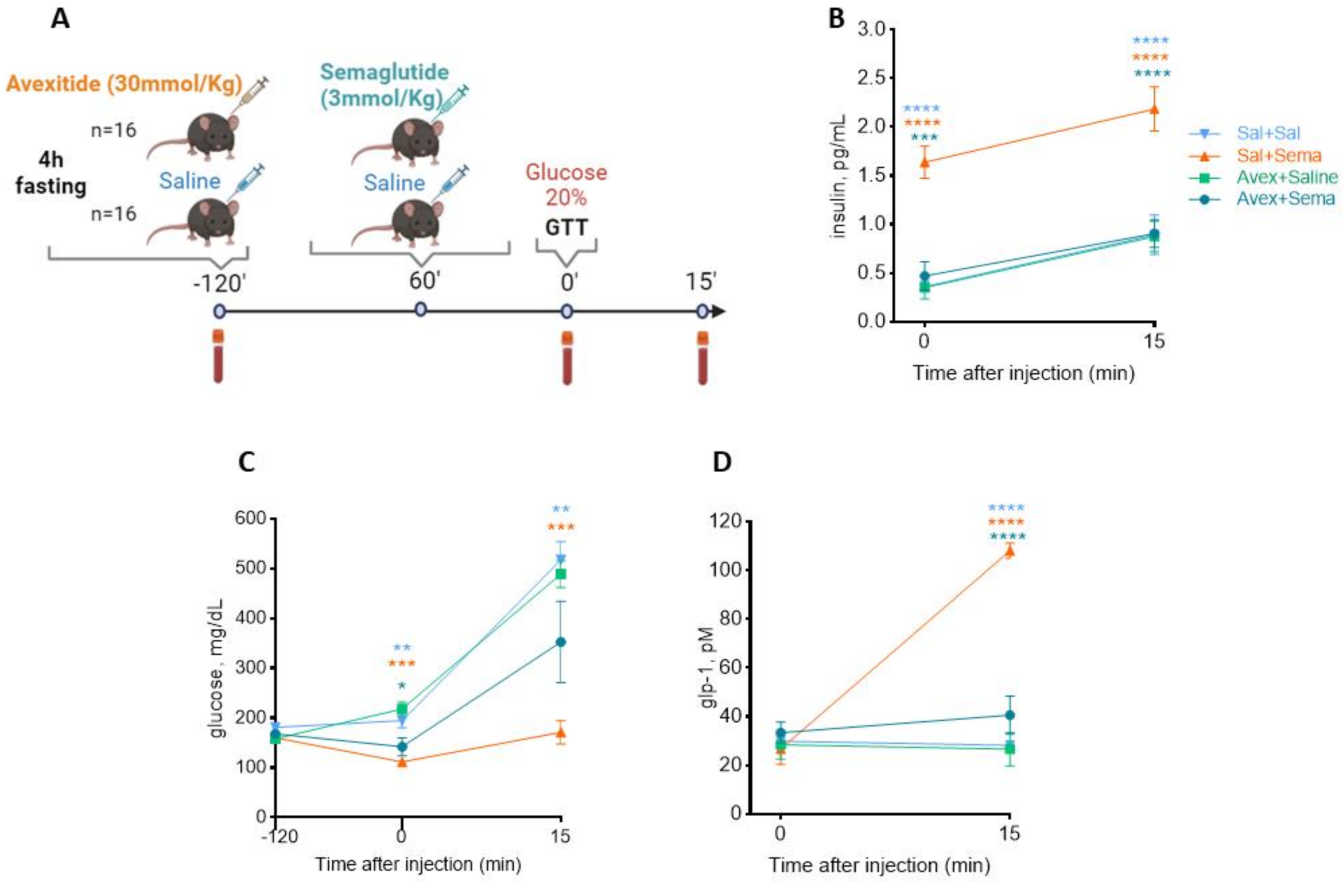

Supplementary Figure 13. GLP-1 receptor antagonist (avexitide) blocks the effects of GLP-1 agonist (semaglutide). (A) Scheme demonstrating the administrations of avexitide (30mmol/Kg) and semaglutide (3mmol/Kg) during the glucose tolerance test. (B) Insulin levels during GTT. (C) Glucose levels during GTT. (D) GlP-1 levels during GTT. In all panels, ***p<0.001, ****p<0.0001 by ANOVA 2-way with Tukey's multiple comparisons test.

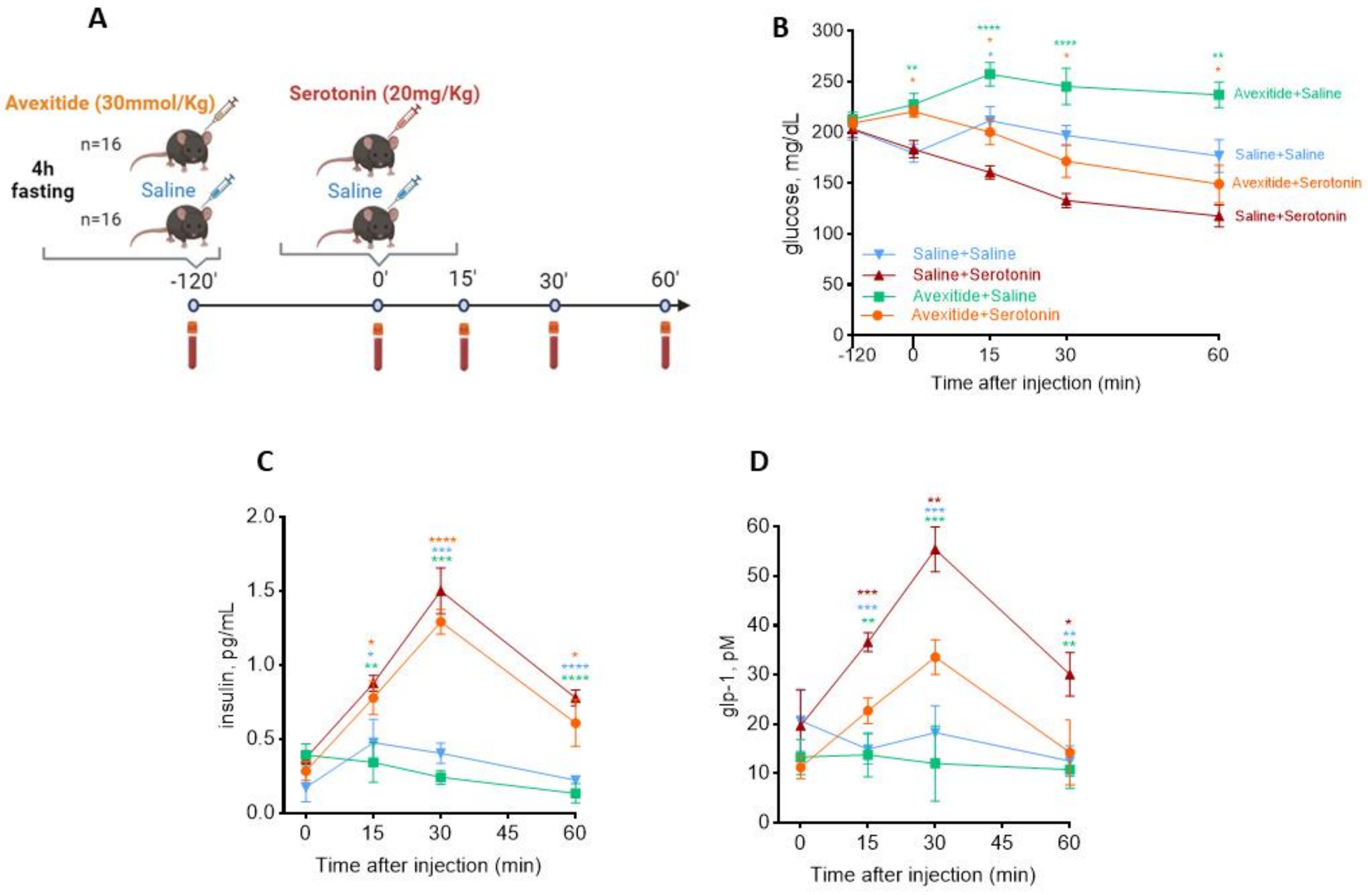

Supplementary Figure 14. Administration of GLP-1 antagonist (avexitide) does not block serotonin-induced hypoglycemia. (A) Scheme demonstrating the administrations of avexitide (30mmol/Kg) and serotonin (20mg/Kg) in C57BL/6J mice. (B) Glucose levels after serotonin or saline injection. (C) Insulin levels, and (D) GLP-1 levels after serotonin or saline injection. ANOVA 2-way with Tukey's multiple comparisons test was performed.

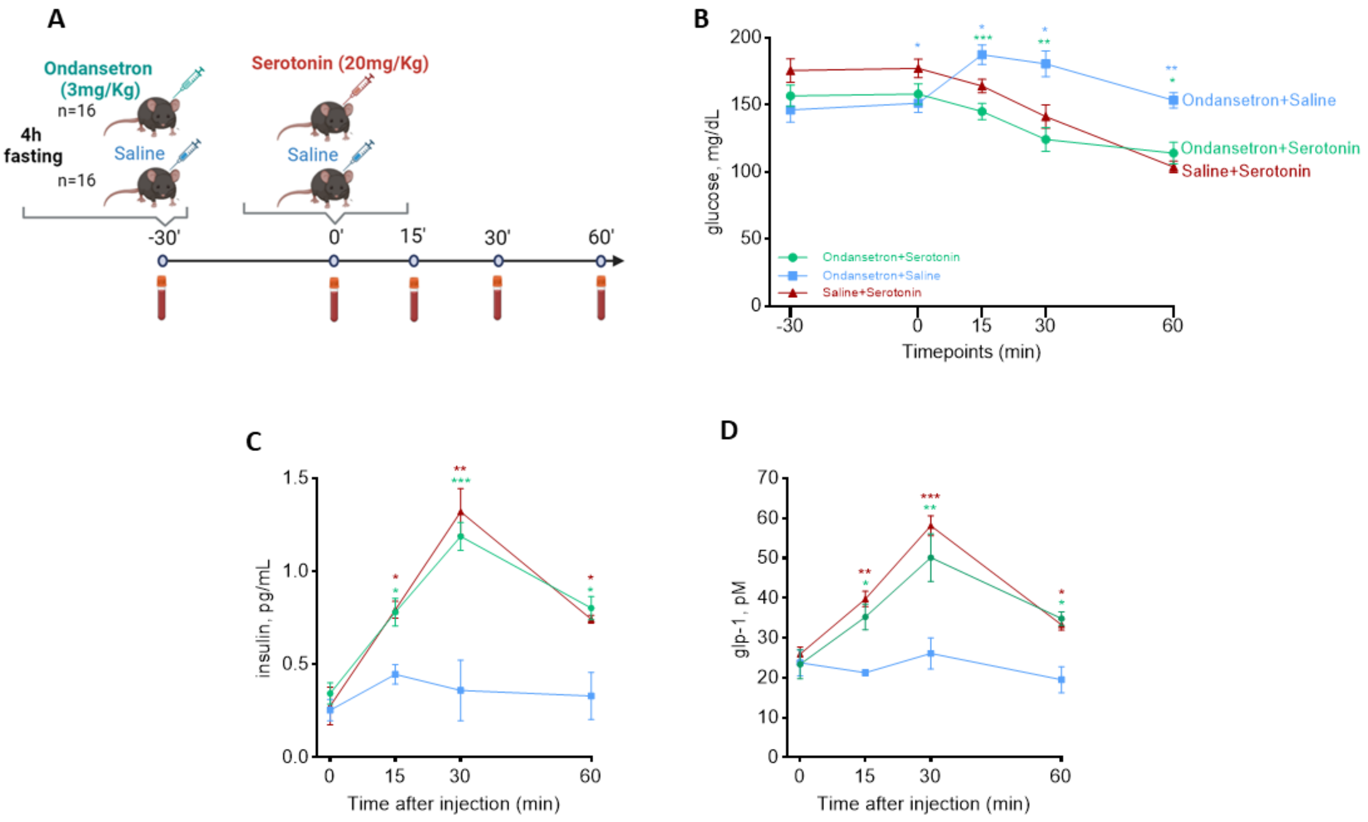

Supplementary Figure 15. Serotonin-induced reduction in glucose is not blocked by the serotonin receptor 3 antagonist ondansetron. (A) Scheme illustrating experiment showing administration of ondansetron (3mg/Kg) and exogenous administration of serotonin (20mg/Kg) or saline in C57BL/6J mice. (B) Glucose levels, (C) Insulin levels, and (D) GLP-1 levels after serotonin or saline injection. In all panels, **p<0.01, ***p<0.001, ****p<0.0001 by ANOVA 2-way with Tukey's multiple comparisons test.

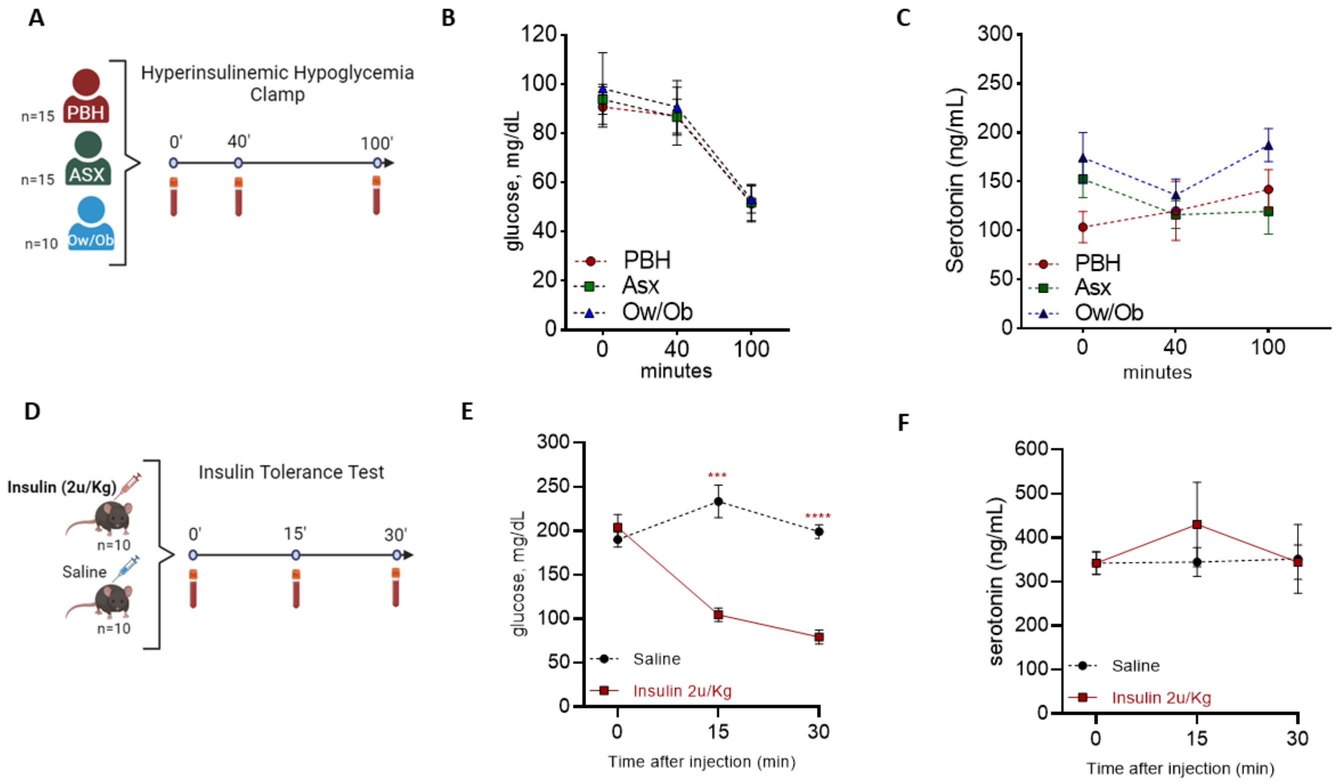

Supplementary Fig. 16. Glucose and serotonin levels during hyperinsulinemic hypoglycemic clamp in PBH, Asx and Ow/Ob individuals and insulin tolerance test using high dose insulin in mice. (A) Schematic of experiment showing hyperinsulinemic hypoglycemic clamp in PBH, Asx and Ow/Ob humans. (B) Glucose and (C) serotonin levels during hyperinsulinemic hypoglycemic clamp. (D) Schematic of insulin tolerance test (ITT, 2U/Kg or saline) in C57BL/6J mice. (E) Glucose and (F) serotonin levels during ITT. In all panels, ***$p<0.001$, ****$p<0.0001$ by ANOVA 2-way with Tukey's multiple comparisons test.

	Surgical		Non-Surgical	
	**Post-bariatric hypoglycemia**	**Asymptomatic**	**Overweight/Obese**	**P-value**
Age (years)	47 ±10	48 ±11	49 ±10	0.99
Gender (M/F)	2/11	2/8	3/5	0.51
White/Black/Declined to specify	12/0/1	3/0/7	5/2/1	-
Non-Hispanic/Latino-Hispanic/Declined to specify	11/1/1	2/1/7	7/0/1	-
Years post-surgery	3.1	2.6	na	0.47
BMI (Kg/m²)	30.6 ±4.9	29.9 ±4.4	41.8 ±10.8	0.004# /0.004$
Waist (cm)	96.2 ±10.2	94.5 ±17.1	124 ±29.7	0.011# /0.011$
Hb1Ac (%)	5.2 ±0.2	5.2 ±0.4	5.4 ±0.3	0.21
Cholesterol (mg/dL)	167.0 ±31.0	176.4 ±26.5	190.8 ±27.6	0.21
Triglycerides (mg/dL)	77.8 ±32.1	50.7 ±12.1	165.6 ±112	0.012# /0.002$
HOMA-IR	0.82 ±0.2	1.04 ±1.2	2.48 ±1.3	0.001# /0.009$
Matsuda index	4.5 ±1.8	4.9 ±2	2.1 ±3.3	0.55

Supplementary Table 1. Clinical characteristics of participants. P values were calculated using one-way ANOVA 1-way with Tukey post-test. *PBH vs. Asx, #PBH vs. Ow/Ob, $Asx vs. Ow/Ob.

	Post-Bariatric Hypoglycemia	Asymptomatic	Overweight/Obese
Age (years)	54±11	53±9	47±13
Gender (M/F)	1/14	0/15	4/6
White/Black/more than one race	15/0/0	14/1/0	6/4
Non-Hispanic or Latino-Hispanic	15/0	13/2	10/0
Years since RYGB (yr)	8±5	11±5	n/a
Weight (kg)	80±9	89±22	95±16
BMI (kg/m2)	30±4	34±7	32±5
Fasting HbA1C (%)	5±0.3	5±0.3	5±0.2
SSRI user	5/15	5/15	1/10
SNRI user	1/15	1/15	0/10

Supplementary Table 5. Clinical characteristics of cohort participants for serotonin ELISA analysis, using blood samples obtained during mixed meal and hyperinsulinemic hypoglycemic clamp.
